# Mitochondria in Viral Infections: Molecular Mechanisms and Therapeutic Targets

**DOI:** 10.1002/mco2.71127

**Published:** 2026-11-05

**Authors:** Ha Young Oh, Hye Jin Shin

**Affiliations:** ^1^ Department of Microbiology Chungnam National University School of Medicine Daejeon Republic of Korea; ^2^ Department of Medical Science Chungnam National University School of Medicine Daejeon Republic of Korea; ^3^ Brain Korea 21 FOUR Project for Medical Science Chungnam National University Daejeon Republic of Korea

**Keywords:** innate immunity, metabolic reprogramming, mitochondria, oxidative phosphorylation (OXPHOS), viral replication

## Abstract

Mitochondria are key regulators of cellular metabolism and innate immunity, and diverse viruses manipulate these organelles during infection. Viral proteins interact with respiratory chain complexes to regulate oxidative phosphorylation (OXPHOS) and reprogram host bioenergetics, thereby influencing viral replication or host antiviral responses. OXPHOS can be directly altered by viral factors or indirectly regulated through virus‐induced changes in host metabolism and signaling. Beyond OXPHOS, viral infection perturbs mitochondrial metabolism, dynamics, calcium homeostasis, membrane potential, redox regulation, and apoptosis, linking mitochondrial alterations to viral replication and virus‐associated cellular responses. This review focuses on how viruses regulate mitochondrial function, with particular emphasis on OXPHOS while addressing other mitochondrial processes in viral infection. We further distinguish mitochondrial alterations functionally linked to viral infection outcomes from those observed during infection without direct evidence of their effects on viral infection outcomes. Mitochondrial pathways have dual functions during infection, as viruses can hijack these pathways to promote infection, whereas hosts can utilize them to support antiviral responses. By integrating these mechanistic and experimental findings, this review highlights virus‐specific patterns of mitochondrial regulation and their functional significance during infection. A mechanistic understanding of these mitochondrial responses provides a basis for identifying mitochondrial pathways as potential antiviral targets.

## Graphical abstract

Viral infections remodel mitochondrial function through direct viral regulation and infection‐induced changes in host cellular processes. These alterations affect OXPHOS, mitochondrial metabolism, calcium homeostasis, membrane potential, redox regulation, dynamics, and apoptosis, with distinct effects on viral replication and host antiviral responses. Mitochondrial regulation can therefore have either proviral or antiviral functions depending on the virus and mitochondrial process involved. Selective modulation of mitochondrial pathways represents a potential antiviral strategy.

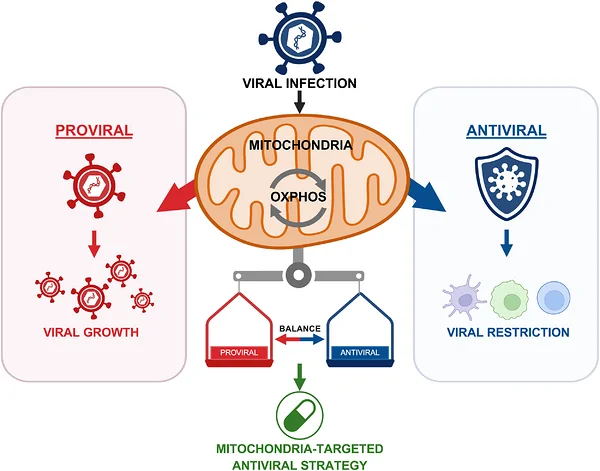

## 1 Introduction

Mitochondria are essential organelles that coordinate cellular energy metabolism, signaling, and programmed cell death. In addition to their classical role in adenosine triphosphate (ATP) production, mitochondria serve as regulatory platforms that coordinate metabolic and innate immune signaling, thereby regulating cellular homeostasis under both physiological and pathological conditions [1, 2, 3, 4]. They are also major targets of viral factors because of their central role in host defense and viral replication [5, 6, 7, 8]. Many viruses encode proteins that localize to mitochondria or mitochondria‐associated membranes, where they directly or indirectly modulate organelle function to promote viral replication and persistence [9, 10].

A major determinant of mitochondrial bioenergetics is the electron transport chain (ETC), composed of complexes I–IV, which transfer electrons from reduced substrates to oxygen, generate a transmembrane proton gradient, and power ATP synthesis through oxidative phosphorylation (OXPHOS) [11, 12]. Under aerobic conditions, OXPHOS provides most cellular ATP, with its activity adjusted to cellular energy demand. Electron transfer through the ETC also generates reactive oxygen species (ROS), linking mitochondrial respiration to both oxidative damage and redox signaling [13, 14, 15]. At controlled levels, ROS regulate redox signaling and immune activation, whereas excessive ROS production induces oxidative stress and cellular damage. ATP generated by OXPHOS is required for multiple energy‐dependent stages of the viral life cycle, including genome replication, nucleocapsid assembly, and particle release. Viral proteins, together with infection‐induced metabolic changes, reprogram mitochondrial activity to supply energy required for viral replication [16, 17, 18, 19]. Viral modulation of OXPHOS is not restricted to increased ATP production; in viral infection, selective suppression of respiratory complexes redirects host metabolism or modifies energy production and innate immune signaling. Mitochondrial respiration is also integrated with antiviral signaling. For example, ROS derived from OXPHOS regulate mitochondrial antiviral signaling (MAVS) dependent and related signaling pathways that control interferon responses [20, 21, 22]. At the mitochondrial outer membrane, MAVS connects viral RNA sensing with downstream interferon production. Viral proteins can remodel mitochondrial membranes, induce or inhibit apoptosis, and modify electron flow through specific complexes to promote viral replication [9, 23, 24, 25]. Mitochondrial calcium regulation, fusion–fission balance, and membrane potential are actively altered during infection, thereby coupling mitochondrial structural remodeling to functional changes [26, 27, 28, 29]. Although OXPHOS is a major target, viruses also reprogram other metabolic pathways, including glycolysis, lipid metabolism, and amino acid turnover to support viral replication and regulate antiviral immune responses [30, 31, 32, 33]. These metabolic changes not only generate substrates required for viral synthesis but also disrupt cellular homeostasis, resulting in apoptosis and altered long‐term physiology [2, 34, 35, 36]. Mitochondrial dysfunction caused by infection therefore alters not only energy metabolism but also calcium regulation, oxidative stress responses, and apoptotic thresholds, thereby affecting homeostasis, aging, and disease progression [1, 2, 3, 37]. These mitochondrial responses differ across viruses and are determined by host cell type, animal model, and stage of infection, demonstrating cell type‐ and stage‐specific differences in virus host interactions [7, 8]. Even within the same respiratory complex, different subunits have distinct functions during viral infection, demonstrating subunit‐specific regulation of virus–mitochondria interactions.

In this review, we summarize current evidence on how viral infection modulates mitochondrial function, with a focus on OXPHOS and its relationship with other mitochondrial processes. We organize the evidence according to two main aspects: (i) the level of mitochondrial involvement, distinguishing OXPHOS‐centered mechanisms from mitochondrial processes beyond OXPHOS, and (ii) the level of functional evidence and experimental approach. Specifically, we distinguish studies that provide direct functional evidence linking mitochondrial regulation to viral genome replication, viral protein expression, or infectious virion production, which supports classification of mitochondrial pathways as proviral or antiviral, from studies based on experimental systems without direct validation of viral infection outcomes. These include experimental approaches that do not represent complete, replication‐competent viral infection capable of producing infectious virions, such as single viral protein expression, transcript‐based constructs, or pseudoviruses, as well as studies performed in diverse host models with varying cellular and physiological conditions. In such cases, mitochondrial alterations are observed, but their effects on viral infection outcomes have not been directly established and therefore do not support classification as proviral or antiviral. Based on this framework, Section 2 focuses on OXPHOS complexes as core bioenergetic regulators, whereas Section 3 addresses mitochondrial functions beyond OXPHOS. This framework enables systematic comparison between functionally validated mechanisms and observations derived from limited or indirect models, clarifying the functional relevance of mitochondrial regulation in virus–host interactions.

## 2 OXPHOS in Viral Infection

Viral infections induce diverse changes in mitochondrial OXPHOS, with consequences that vary according to the virus and experimental conditions. This section specifically examines alterations in OXPHOS complexes and their components by distinguishing changes that have been functionally linked to viral infection outcomes from those observed during infection for which the functional significance of the specific OXPHOS alteration remains incompletely defined. Functional outcomes include viral genome replication, viral protein expression, infectious virion production, viral persistence, cytopathogenicity, and other virus‐associated cellular outcomes. In this review, proviral effects broadly refer to OXPHOS or mitochondrial processes that contribute to the establishment or progression of these viral infection outcomes, whereas antiviral effects refer to processes that restrict viral infection or promote host antiviral responses. This framework provides a basis for evaluating both the functional significance and infection‐associated patterns of OXPHOS regulation.

### 2.1 OXPHOS Roles in Viral Infection

As shown in Figure 1, viral infection induces distinct, virus‐specific changes in OXPHOS activity, and these alterations can influence viral infection depending on viral factors and the stage of infection. In this section, we focus on studies that examine OXPHOS complexes or their components as core bioenergetic regulators and provide functional evidence linking specific OXPHOS alterations to the viral infection outcomes defined above. As summarized in Table 1, OXPHOS complexes can have either proviral or antiviral roles when their functional effects on viral infection are supported by experimental evidence. The following section examines how these functionally linked OXPHOS alterations contribute to viral infection across diverse viruses.

**FIGURE 1:**
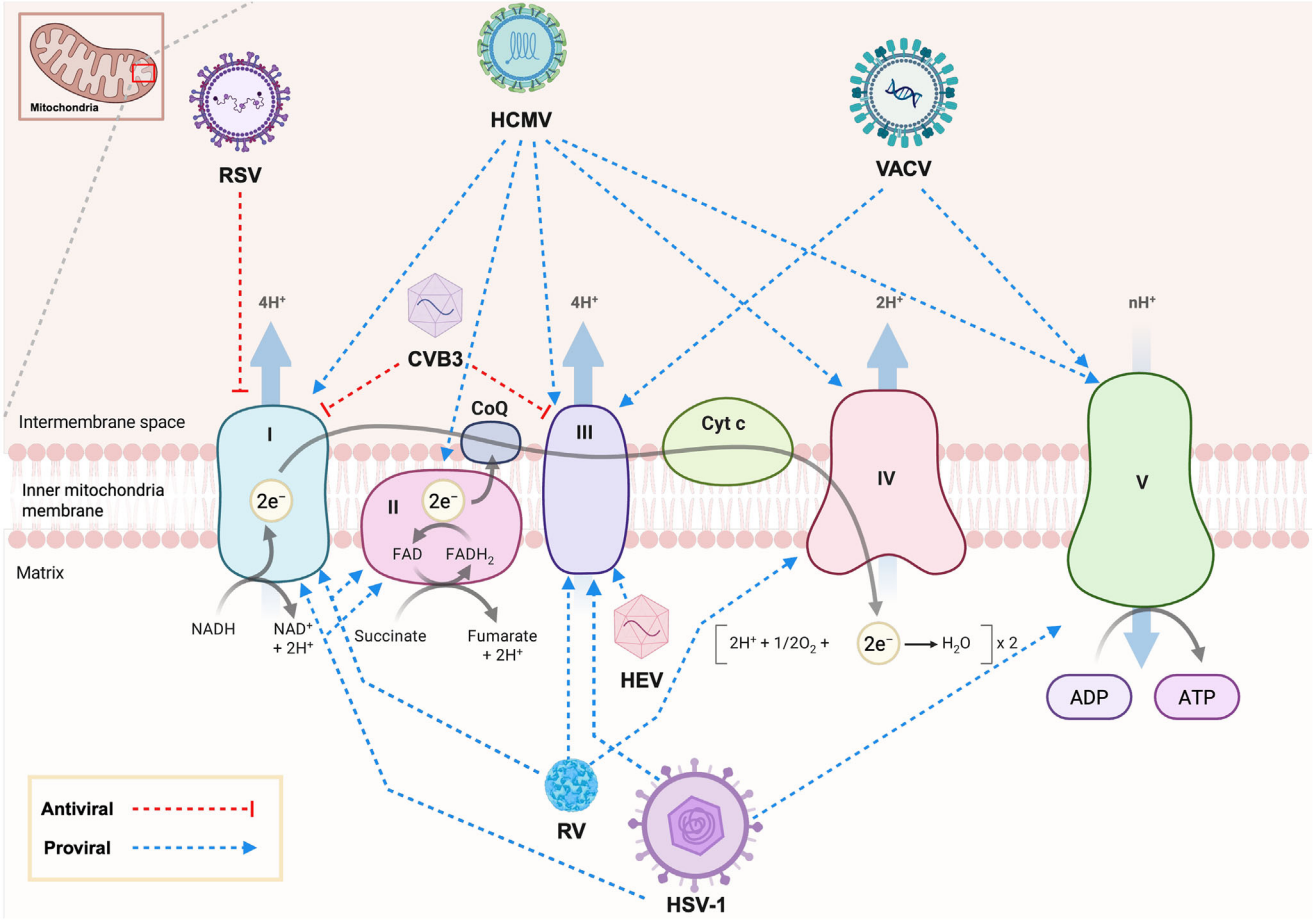
Proviral and antiviral roles of OXPHOS complexes across viral infections. Mitochondrial OXPHOS complexes (I–V) are dynamically regulated in a virus‐specific manner and have either proviral or antiviral effects. Downregulation or knockout of complex I enhances RSV replication, establishing an antiviral role for complex I. Increased activity of complexes I and III suppresses CVB3 titers, indicating antiviral functions. In contrast, HCMV infection is associated with upregulation of complexes I‐V and requires intact mtDNA, reflecting a proviral dependence on OXPHOS. VACV infection enhances mitochondrial respiration, while inhibition of complex V or mitochondrial uncoupling reduces viral yield, confirming a requirement for OXPHOS. HSV‐1 replication similarly requires complexes I, III, and V, with the complex V subunit beta (ATP5B) contributing to viral propagation. Complex III has a proviral function in both RV and HEV infection: its inhibition reduces viral RNA and particle production in RV and suppresses HEV replication. Overall, the functional role of individual OXPHOS complexes differs according to the virus and the specific complex involved.

**TABLE 1:** OXPHOS changes functionally linked to viral infection.

Virus genome	Virus	Effect on viral infection	Viral protein	OXPHOS complex/component	Experimental model	Experimental intervention	Functional outcome	References
RNA	RV	Proviral		Complex III	Vero cell line	Antimycin A (20 µM)	Complex III supports RV replication	[43]
				Complex I, II, IV	A549, MCF‐7, Vero cell lines	Antimycin A (0.0125 mM)	Complex II activation accompanies RV replication	[44]
	CVB3	Antiviral		Complex I, III	C57BL/6, A.SW/SnJ mice		Enhanced OXPHOS associates with CVB3 clearance	[51]
	HEV	Proviral		Complex III	Huh‐7.5, U87 cell lines	Antimycin A (0.1 µM), Myxothiazol (0.1 µM)	Complex III essential for HEV replication	[54]
	RSV	Antiviral		Complex I (NDUFA10, NDUFB10)	HEK293T ([FB4^−/−^], [FB10^−/−^], [FA10^−/−^]) cell lines		Complex I subunit loss enhances RSV replication	[61]
			M	Complex I (NDUFV1, NDUFC2, NDUFA10, NDUFB10)	Vero, A549 cell lines		M‐dependent respiratory remodeling supports RSV production	[62]
DNA	HCMV	Proviral		Complex I, II, III, IV, V	HF cell line		OXPHOS induction accompanies HCMV replication	[68]
				Complex V (ATP5F1A)	HFFF2, U373 cell lines		ATP‐generating capacity supports HCMV replication	[69]
			pUL37×1	Complex IV	Human embryonic fibroblasts		OXPHOS biogenesis supports viral production	[70]
				Complex I (NDUFB8), II (SDHD), III (UQCRC2), IV (COX2)	HFF‐Rho cell line		HCMV replication requires intact mtDNA OXPHOS	[72]
			pUL13	Complex I, IV, V	MRC5 cell line		UL13 deletion lowers respiration, HCMV replication	[73]
	VACV	Proviral		Complex V	HeLa, Huh‐7 cell lines	Oligomycin (4.5 µM)	ATP synthase inhibition reduces VACV replication	[75]
				Complex III	HeLa cell line	Antimycin A (20 µM), CCCP (1 µM)	Complex III blockade suppresses VACV replication	[77]
	HSV‐1	Proviral		Complex I (NDUFB3), II (SDHB), III (CYC1), IV	Patient UCMSCs samples	Rotenone (0.572 µM)	Complex I inhibition suppresses HSV‐1 replication	[78]
				Complex V (ATP5B)	HeLa cell line	shATP5B	ATP5B promotes HSV‐1 replication	[80]

Abbreviations: Virus: RV = Rubella virus, CVB3 = Coxsackievirus B3, HEV = Hepatitis E virus, RSV = respiratory syncytial virus, HCMV = human cytomegalovirus, VACV = vaccinia virus, HSV‐1 = Herpes simplex virus type 1. viral protein: M = matrix protein, pUL13 = protein unique long region 13, pUL37×1 = protein unique long region 37 exon 1. Experimental Model: Vero = African green monkey kidney epithelial cells, A549 = alveolar epithelial cells; human lung carcinoma epithelial cells, MCF‐7 = human breast; mammary gland epithelial cells, C57BL/6 = C57 black 6 mice, A.SW/SnJ = A/WySn; H2^s^ haplotype, including H2‐T18^b^ mice, Huh‐7.5 = human hepatoma‐derived carcinoma cells, U87 = human brain likely glioblastoma cells, HEK293T = human embryonic kidney cells with simian virus 40 large T‐antigen, HEK293T[FB4^−/−^] = HEK293T complex I β subcomplex subunit 4 knock out cells, HEK293T[FB10^−/−^] = HEK293T complex I β subcomplex subunit 10 knock out cells, HEK293T[FA10^−/−^] = HEK293T complex I α subcomplex subunit 10 knock out cells, HFF‐Rho = human foreskin fibroblast cells; devoid of mitochondrial DNA, MRC5 = Medical Research Council cell strain 5; human fetal lung fibroblast cells, HF = primary human foreskin fibroblasts, HFFF2 = primary human fetal foreskin fibroblast cells, U373 cells = human brain; glioblastoma astrocytoma cells, HeLa = human adenocarcinoma epithelial cells, Huh‐7 = human hepatoma‐derived carcinoma cells, UCMSCs = umbilical cord mesenchymal stem cells. Experimental intervention: CCCP = carbonyl cyanide *m*‐chlorophenyl hydrazone.

#### 2.1.1 RNA Viruses

##### 2.1.1.1 Rubella Virus (RV)

Replication of positive‐sense RNA viruses frequently occurs within membrane‐associated compartments that coordinate viral RNA synthesis and assembly. In Rubella virus (RV), this process is organized within cytoplasmic cytopathic vacuoles formed following endocytic entry, where replication and budding are linked to intracellular membranes, including the Golgi apparatus [38, 39, 40, 41]. RV infection modifies mitochondrial bioenergetics in a time‐ and complex‐specific manner. In RV‐infected Vero cells (African green monkey kidney epithelial cells), a highly permissive model for viral propagation, inhibition of cytochrome c reductase (complex III) by antimycin A (AMA) reduces intracellular viral RNA and infectious particle production, with complex III inhibition producing the greatest reduction among the tested ETC complexes [42, 43]. Complex III activity increases relative to mock‐infected controls, and this increase is further enhanced by cytochalasin D. Mitochondrial clustering around replication sites is also observed during viral genome synthesis and assembly. Across Vero, MCF‐7 (human breast adenocarcinoma), and A549 (human lung epithelial) cells, RV infection is associated with elevated mitochondrial membrane potential (ΔΨm) and ATP production, while overall respiratory chain activity is maintained during the replication cycle. Individual respiratory complexes show differential changes, with succinate dehydrogenase (SDH, complex II) showing the most prominent changes, whereas complexes I and IV show smaller changes in a cell line‐dependent manner [44].

##### 2.1.1.2 Coxsackievirus B3 (CVB3)

Coxsackievirus B3 (CVB3), a member of the Enterovirus genus within the Picornaviridae family, is a positive‐sense single‐stranded RNA virus that replicates in the cytoplasm within virus‐induced replication organelles derived from host intracellular membranes. In enteroviruses including CVB3, the membrane‐associated replication structures facilitate viral RNA synthesis and organize multiple steps of the viral replication cycle [45, 46, 47, 48]. CVB3 is a major etiologic agent of viral myocarditis (MC). The A.SW/SnJ mouse strain develops chronic myocarditis following persistent viral infection, and this model reproduces key pathological features of the pathophysiological features observed in human CVB3‐induced MC, including immune cell infiltration, myocardial inflammation, and long‐term tissue remodeling [49, 50]. To determine whether modulation of mitochondrial bioenergetics alters cardiac viral burden in MC, A.SW/SnJ mice were infected with CVB3. Analysis of infected cardiac tissues reveals an average reduction in the enzymatic activity of all respiratory chain complexes compared with uninfected controls. Reduced myocardial viral titers are strongly associated with enhanced activities of NAD ubiquinone oxidoreductase (complex I) and complex III, suggesting a potential inverse relationship between mitochondrial respiratory function and CVB3 replication in heart tissue [51].

##### 2.1.1.3 Hepatitis E Virus (HEV)

Hepatitis E virus (HEV) is a positive‐sense single‐stranded RNA virus that replicates in the cytoplasm of infected cells, where viral genome replication and subgenomic RNA translation occur during the viral replication cycle. Beyond hepatic infection, HEV is associated with extrahepatic manifestations, including neurological complications. Human hepatoma‐derived Huh7 cells support HEV RNA replication and are commonly used to study HEV replication. In addition, neuronal cell models, including human glioblastoma‐derived U87 cells, support HEV replication and production of infectious viral particles [52, 53]. In the Huh7.5‐p6 luciferase reporter system, short‐term exposure to mitochondrial complex III inhibitors, AMA and myxothiazol (MYXO), reduces HEV replication. AMA and MYXO also reduce HEV replication in U87 cells harboring an HEV subgenomic replicon, demonstrating complex III dependence in both hepatic and neuronal cell models. Viral RNA levels decrease following inhibitor treatment, together with reduced viral protein expression. These findings demonstrate that mitochondrial complex III activity supports HEV replication. Complex III inhibition also alters ΔΨm and ATP production, linking respiratory function to the metabolic requirements of viral RNA synthesis [54].

##### 2.1.1.4 Respiratory Syncytial Virus (RSV)

Respiratory syncytial virus (RSV), a negative‐sense single‐stranded RNA virus of the Pneumoviridae family, replicates in the cytoplasm of infected cells through the formation of viral inclusion bodies, where viral RNA synthesis and nucleocapsid formation occur. RSV primarily infects airway epithelial cells in the respiratory tract [55, 56]. Human lung epithelial A549 cells support RSV infection and provide an established in vitro model for studying RSV host interactions [57, 58]. RSV infection induces microtubule‐ and dynein‐dependent repositioning of mitochondria toward the microtubule‐organizing center in A549 cells, leading to reduced mitochondrial respiration and increased mitochondrial reactive oxygen species (mtROS) [59, 60]. Disruption of mitochondrial complex I further increases mtROS production. Knockout (KO) of NDUFA10 or NDUFB10, critical subunits of mitochondrial complex I, leads to increased mtROS levels and enhanced RSV replication. NDUFB10 KO cells produce significantly higher levels of infectious RSV particles compared with wild‐type or NDUFB4 KO cells. NDUFA10‐ and NDUFB10‐deficient cells show increased viral genome copy numbers and infectious particle production relative to wild‐type and reconstituted counterparts, supporting a functional relationship between complex I integrity and antiviral restriction [61]. At the transcriptional level, RSV infection selectively suppresses the expression of genes encoding complex I subunits, including NDUFA10, NDUFB10, NDUFV1, and NDUFC2, without affecting housekeeping gene expression. This transcriptional suppression depends on the RSV M protein, with R170 and K172 within its central nucleic acid‐binding domain required for this regulatory effect. Recombinant RSV carrying R170T/K172T substitutions fails to induce the mitochondrial gene suppression observed with wild‐type RSV. This downregulation is reversed by mitoquinone mesylate (MitoQ), a mitochondria‐targeted ROS scavenger, demonstrating that mtROS contributes to the suppression of complex I gene expression during infection [62]. These results indicate that RSV manipulates mitochondrial dynamics and redox signaling to impair complex I function, thereby promoting RSV replication.

#### 2.1.2 DNA Viruses

##### 2.1.2.1 Human Cytomegalovirus (HCMV)

During productive infection, human cytomegalovirus (HCMV), a large double‐stranded DNA (dsDNA) virus of the Herpesviridae family, undergoes a gene expression cascade consisting of immediate‐early (IE), early (E), and late (L) phases, with viral DNA replication and nuclear capsid assembly occurring in the nucleus [63, 64]. HCMV infection reprograms host cellular metabolism, particularly mitochondrial bioenergetics, to support viral replication. Primary human fibroblasts are commonly used to study HCMV infection [65, 66, 67]. Transcriptomic and proteomic analyses show increased expression of genes encoding ETC components, including components of complexes I–IV, multiple ATP synthase (F_0_F_1_‐ATPase, complex V) subunits, and factors involved in respiratory complex assembly and regulation [68, 69]. Functional assays show increased mitochondrial biogenesis and elevated oxygen consumption rates (OCRs) during infection. Mitochondrial protein synthesis is required for the increase in respiratory capacity, as inhibition with chloramphenicol prevents this respiratory increase and significantly reduces viral replication. At the viral protein level, pUL37×1 contributes to the HCMV‐induced increase in mitochondrial respiration, as deletion of UL37×1 significantly attenuates the infection‐associated increase in respiratory capacity. This regulation is accompanied by sustained mtROS production, which is also reduced during infection with the ΔUL37×1 virus, demonstrating a role for pUL37×1 in the coordinated regulation of mitochondrial respiration and redox status during HCMV infection [70]. Human foreskin fibroblast (HFF) ρ^0^ cells, which lack mitochondrial DNA (mtDNA) encoded ETC components, show markedly reduced viral replication [71]. At the level of individual ETC components, HCMV infection induces differential changes in protein abundance. In wild‐type HFFs, infection increases the complex I subunit (NDUFB8) but more strongly elevates the complex II subunit (SDHD), complex III subunit (UQCRC2), and cytochrome c oxidase (complex IV) subunit (COX2) at early time points postinfection. In contrast, these changes are absent in ρ^0^ cells, which also show reduced viral replication, as measured by IE protein expression [72]. HCMV viral proteins also regulate mitochondrial bioenergetics. The HCMV protein pUL13 regulates the abundance of multiple OXPHOS complexes, and its deletion results in reduced levels of complexes I, IV, and V, decreased basal and ATP‐linked respiration, and reduced viral titers. Mitochondria‐localized pUL13 associates with components of ETC complexes II–IV and with the protein inhibitor of complex and leucine‐rich pentatricopeptide repeat containing protein (LRPPRC), which support ETC function and mitochondrial protein expression, respectively. pUL13 also interacts with components of the mitochondrial contact site and cristae organizing system (MICOS), including inner membrane mitochondrial protein (IMMT) and coiled‐coil‐helix‐coiled‐coil‐helix domain‐containing protein 3 (CHCHD3), which regulate mitochondrial cristae architecture and OXPHOS organization [73]. The HCMV protein pUL37×1 influences mitochondrial function, with its absence leading to a twofold reduction in replication, and its absence combined with chloramphenicol treatment causing further viral suppression [70]. These studies demonstrate that HCMV infection activates OXPHOS gene expression, enhances ETC complex abundance and assembly, and increases mitochondrial respiratory activity, with pharmacological or genetic impairment of OXPHOS consistently suppressing viral replication.

##### 2.1.2.2 Vaccinia Virus (VACV)

Unlike nuclear‐replicating DNA viruses, Vaccinia virus (VACV), a prototypical member of the Poxviridae family, is a large dsDNA virus that completes its entire replication cycle in the cytoplasm, forming viral factories where viral DNA replication, transcription, and virion assembly occur independently of the host nucleus [74]. To determine whether the increased ATP production after VACV infection is necessary for virus production, oligomycin, a mitochondrial complex V inhibitor that blocks ATP synthesis, is used. No amplification of mtDNA after VACV infection is observed, suggesting that ND4 upregulation occurs through transcriptional regulation. Four DNA fragments with altered expression are identified following VACV infection. Two of these fragments correspond to VACV genes, whereas the other two correspond to the mitochondrial genes ND4 and COX2. Induction and upregulation of ND4 expression by VACV infection are confirmed in both HeLa and HuH7 cells. The expression of two viral proteins (A‐type inclusion protein and IMV heparin‐binding surface protein) is suppressed in the presence of oligomycin [75]. VACV infection increases oxygen consumption, indicating increased mitochondrial respiratory activity and OXPHOS activity. This metabolic reprogramming is characterized by increased glutamine utilization and accumulation of energy‐related intermediates [76]. Pharmacological disruption of OXPHOS using carbonyl cyanide m‐chlorophenylhydrazone (CCCP, uncoupler) or AMA (complex III inhibitor) at noncytotoxic concentrations reduces VACV replication, establishing mitochondrial respiration as a requirement for efficient VACV replication [77].

##### 2.1.2.3 Herpes Simplex Virus Type 1 (HSV‐1)

In umbilical cord‐derived mesenchymal stem cells (UCMSCs) infected with Herpes simplex virus type 1 (HSV‐1), transcripts encoding multiple OXPHOS components (NDUFB3, SDHB, and CYC1) change in parallel with transcription factors involved in mitochondrial biogenesis. During infection, complex I and III proteins are increased during the early stage of infection and subsequently decreased, reflecting a dynamic regulation of mitochondrial respiration. Pharmacological inhibition of complex I with rotenone or glycolysis with iodoacetic acid each reduces HSV‐1 replication, and the combination treatment produces the most pronounced effect. Both inhibitors, either individually or together, suppress viral gene transcripts (UL54, UL52, UL27) and early viral proteins (ICP0, ICP8) within 6 h postinfection (hpi) [78]. Complex V activity is required for efficient HSV‐1 replication, including viral DNA encapsidation and capsid maturation [79]. Bioinformatic analysis identifies a predicted miR‐101 binding site in the 3′UTR of the mitochondrial complex V subunit beta (ATP5B), and miR‐101 directly targets ATP5B to suppress its expression. RNAi‐mediated silencing of ATP5B reduces its endogenous levels and results in smaller HSV‐1 plaques in HeLa cells without affecting cell viability, supporting a proviral role for ATP5B. Overexpression of miR‐101 also inhibits HSV‐1 replication [80].

### 2.2 OXPHOS Changes during Viral Infection

Table 2 summarizes alterations in OXPHOS complexes, their components, or mitochondrial respiration observed during viral infection for which the functional significance of the specific OXPHOS alteration remains incompletely defined. Although these studies identify OXPHOS‐related changes under different infection conditions, they do not establish a functional link between the specific OXPHOS alteration and the viral infection outcomes defined above. Therefore, these OXPHOS changes cannot be definitively classified as proviral or antiviral and are instead interpreted as alterations associated with viral infection. This section highlights OXPHOS changes whose functional significance in viral infection remains to be established.

**TABLE 2:** OXPHOS changes during viral infection.

Virus Genome	Virus	Viral protein	OXPHOS complex/component	Experimental model	Experimental intervention	Observed OXPHOS changes	References
RNA	HCV	Core	Complex I	SL‐139 mice	Rotenone (1 µM)	HCV core elevates complex I‐associated ROS	[85]
			Complex I	UHCVcon‐57.3 cell line	Antimycin A (1 µg/mL), KCN (2 mM), Rotenone (2 µg/mL)	Complex I restoration reduces HCV‐induced Ca^2^^+^ overload	[86]
			Complex I, V	UHCV‐32 cell line	Antimycin A (1 µg/mL), KCN (2 mM), Rotenone (2 µg/mL)	Complex V activity recovers during HCV infection	[87]
		p7 (ATP5H) NS4B (ATP6)	Complex V (ATP5H, ATP6)	Huh‐7, HEK293T cell lines		HCV proteins target ATP synthase	[88]
			Complex I (ND1, ND3, ND4, ND4L), IV (COX2)	Huh‐7.5 cell line		Complex I/IV downregulation occurs during HCV persistence	[90]
			Complex I, V	UHCVcon‐57.3 cell line	Antimycin A (1 µg/mL), KCN (2 mM), Rotenone (2 µg/mL), CCCP (1 µM)	HCV suppresses complex I/V while maintaining ATP	[91]
	ZIKV		Complex II	C57BL/6NJ ([Ripk3^−/−^], [Irg1^−/−^]) mice	Dimethyl malonate (1 mM), Lonidamine	ZIKV infection increases SDH activity and ATP	[94]
			Complex I (NDUFS3, NDUFA4, NDUFA5), IV (COX4I1, COX6C), V (ATP8)	ICR mice		Neuronal OXPHOS downregulation associates with ZIKV pathology	[95]
	WNV		Complex II (SDHC), IV (COX5B, COX6B), V (ATP5G1, ATP5C1, ATP5J, ATP5B, ATP5F1A, ATP5O, ATP5F1)	A172 cell line		WNV downregulates ETC subunits and lowers energy	[98]
			Complex IV (COX6B2)	Hsd:ICR(CD‐1) mice		WNV decreases complex IV subunit Cox6b2 expression	[99]
	SARS‐CoV‐2		Complex I, II, III, IV, V	HEK293T, Calu‐3 cell lines K18‐hACE2 transgenic mice		Early SARS‐CoV‐2 infection upregulates OXPHOS	[102]
			Complex I (NDUFV1), II (SDHA), III (COR1, UQCRC2), V (ATP5J, ATP5F1A, ATP5H, F‐type ATPase α)	Patient monocyte samples	Oligomycin D (1 µM), Rotenone (500 nM), Dimethyl malonate (10 mM)	Early SARS‐CoV‐2 infection increases OXPHOS	[103]
			Complex I, II, III, IV, V	Patient nasopharyngeal samples		OXPHOS genes decrease under high viral load	[104]
			Complex I (NDUF)	A549, A549‐ACE2, Calu‐3 NHBE cell lines		Complex I downregulation reduces ROS during infection	[105]
		ORF3c	Complex III (CYTB), IV (COX3)	HSAEC1‐KT cell line		hORF3c upregulates complex III/IV genes	[106]
			Complex I (NDUFB8, NDUFC2, NDUFA11)	Patient blood samples		COVID‐19 CD8^+^ T cells downregulate OXPHOS	[108]
			Complex III (CYTB), Complex V (ATP6)	Patient blood samples		Complex III/V variants associate with reduced ATP	[111]
			Complex I (ND4, ND4L, ND5, ND2), Complex IV (COX1)	Patient blood samples		Complex I/IV variants associate with impaired respiration	[112]
		N	Complex I, III	A549, HBE cell lines		SARS‐CoV‐2 upregulates complex I/III despite ATP loss	[115]
	Influenza virus		Complex IV (COX4I1)	MDCK, HEK293T, COX4I1‐KO‐1 cell lines	Lycorine (0.52 µM)	H5N1 infection upregulates COX4I1	[119]
			Complex IV (COX6C)	A549 cell line	miRNA‐4276 (25 nM)	H1N1 induces miR‐4276‐mediated COX6C suppression	[120]
			Complex I	C57BL/6JRj mice		H3N2 downregulates complex I genes	[121]
	RABV		Complex I, IV	PC12, MNA cell lines Sprague–Dawley rat	KCN (1 mmol/L), Rotenone (25 µmol/L), Antimycin (1 µg/mL)	Complex I activity correlates with RABV susceptibility	[123]
		P, M	Complex I	HEK293T, MNA cell lines	KCN (1 mM), Rotenone (25 µM)	P increases complex I; M reduces its activity	[124]
			Complex I (NDUFB8), III (UQCRC2), II, IV, V	Patient brain samples, Brain canine		RABV alters OXPHOS independently of viral burden	[125]
	Mokola virus	M	Complex IV (COX1)	HeLa cell line		M‐MOK binds complex IV, reduces activity, induces apoptosis	[126]
	HIV		Complex II, III, IV	Patient PBMC samples	Malonate (10 mM), Antimycin A (1 µM), KCN (0.24 mM)	HIV reduces mtDNA and complex II–IV activity	[129]
			Complex IV (COX1)	Patient peripheral nerve samples Macaca nemestrina, Macaca fascicularis macaques		HIV/SIV neuropathy reduces mitochondrial proteins and COX1	[130]
			Complex I (NDUFA6)	Patient PBMC samples CEM‐GFP, Jurkat cell lines	Rotenone (3 µM)	HIV‐1 downregulates NDUFA6 and complex I activity	[131]
			Complex I, II, III, IV, V	CEM‐GFP, Jurkat cell lines	Malonate (10 mM), Antimycin A (0.4 µM)	Complex IV increases; complex I–III decrease in HIV‐1	[132]
			Complex III, V	Patient blood samples	Antimycin A (1 uM), Oligomycin (1 µM)	HIV infection increases glutamine‐dependent OXPHOS	[134]
			Complex I, III	Patient PBMC samples, Jurkat cell line NRG mice	Rotenone (1 µM), Metformin (5 mM), Antimycin A (1 µM)	HIV‐1 infection alters complex I/III activity	[135]
		Vpr	Complex I, II, III, IV	DY150		Vpr broadly inhibits OXPHOS activity	[136]
		p2 Peptide	Complex IV	Patient PBMC samples, MAGIC‐5, MT‐4 cell lines		HIV‐1 p2 peptide enhances complex IV activity	[137]
		Tat	Complex IV	Patient tissue samples, BALB/c mice		Tat inhibits complex IV activity	[138]
	DENV	NS3pro	Complex I	Huh‐7 cell line	Rotenone (10 µM)	NS3 protease impairs complex I activity	[139]
DNA	HBV		Complex I, II, IV	Patient liver samples		HBV lowers nuclear OXPHOS genes, not mtDNA	[144]
		HBx	Complex IV (COX3)	HL‐7702 cell line		HBx elevates COX3 and COX activity	[145]
		HBx	Complex I, III, IV, V	HepG2 cell line	Rotenone (1.25 µm), TTFA (12.5 µm), Antimycin A (12.5 µm), Azide (500 µm), Oligomycin B (6.25 µm)	HBx suppresses multiple OXPHOS complexes	[147]
	EBV		Complex I, III	Patient blood samples	Piericidin A (1 µM), Antimycin A (1 µM)	EBV infection enhances OXPHOS in B cells	[151]
		LMP1	Complex I (ND6, NDUFB8), II (SDHB), III (UQCRC2), IV (COX2), V (ATP6, ATP5F1A)	CNE1, C666‐1 cell lines		LMP1 represses OXPHOS via DNMT1 methylation	[152]
	HPV	E2	Complex III (UQCRC2, UQCRFS1, CYC1), IV (COX2), V (ATP5O)	HaCaT cell line		HPV E2 elevates ROS, impairing OXPHOS	[157]
		E7	Complex V (ATP5B)	C33‐A, HEK293 cell lines		HPV E7 interacts with complex V ATP5B	[158]

Abbreviations: Virus: HCV = Hepatitis C virus, ZIKV = Zika virus, WNV = West Nile virus, SARS‐CoV‐2 = severe acute respiratory syndrome coronavirus 2, RABV = Rabies virus, HIV = human immunodeficiency virus, DENV = Dengue virus, HBV = Hepatitis B virus, EBV = Epstein–Barr virus, HPV = human Papillomavirus. Viral protein: Core = capsid protein, p7 = protein 7, NS4B = nonstructural protein 4B, ORF3c = open reading frame 3c, N = nucleocapsid protein, P = phosphoprotein, M = matrix protein, Vpr = viral protein R, p2 peptide = Gag spacer peptide 1, Tat = trans‐activator of transcription, NS3pro = N‐terminal trypsin‐like serine protease domain of nonstructural protein 3, HBx = HBV X protein, LMP1 = latent membrane protein 1, E2 = early protein 2, E7 = early protein 7. Experimental model: SL‐139 = C57BL/6J; HCV structural protein expressing mice, C57BL/6J = C57 black 6 Jackson Laboratory; mice, UHCVcon‐57.3 = U‐2 OS human osteosarcoma‐derived tetracycline‐regulated cells; inducibly expressing the HCV polyprotein, U‐2 OS = human osteosarcoma‐derived cell line, UHCV‐32 = U‐2 OS human osteosarcoma‐derived tetracycline‐regulated cells; inducibly expressing the HCV polyprotein, PBMC = peripheral blood mononuclear cells, Huh‐7.5 = human hepatoma‐derived carcinoma cells, Huh‐7 = human hepatoma‐derived carcinoma cells, C57BL/6NJ = C57 Black 6 NIH Jackson Laboratory; mice, C57BL/6NJ[Ripk3^−/−^] = C57BL/6NJ receptor interacting protein kinases‐1 knock out mice, C57BL/6NJ[Irg1^−/−^] = C57BL/6NJ immunoresponsive gene 1 knock out mice, ICR = Institute of cancer research; mice, A172 = human brain glioblastoma cells, Hsd:ICR(CD‐1) = Harlan Sprague–Dawley: Institute of cancer research (CD‐1); mice, A549 = alveolar epithelial cells; human lung carcinoma epithelial cells, A549‐ACE2 = angiotensin‐converting enzyme 2 ‐expressing A549 cells, Calu‐3 = human lung epithelial Cells, NHBE = normal human bronchial epithelial cells, HSAEC1‐KT = human lung; small airway epithelial cells, Vero E6 = African green monkey kidney epithelial cells, HEK293T = human embryonic kidney cells with simian virus 40 large T‐antigen, K18‐hACE2 transgenic mice = C57BL/6J; express human ACE2, HBE = human lung; Bronchus epithelial cells, MDCK = Madin–Darby canine kidney; cocker spaniel; dog kidney epithelial cells, COX4I1‐KO‐1 = HEK293T COX4I1 Knock out cells, C57BL/6JRj = black 6 Jackson Laboratory Janvier Labs; mice, PC12 = Rattus norvegicus; rat Adrenal gland Pheochromocytoma cells, MNA = mouse neuroblastoma cells, HeLa = human adenocarcinoma epithelial cells, Jurkat = human CD4+ T lymphoblast cells, NRG = NOD‐Rag1null IL2rgnull; mice, DY150 = *Saccharomyces cerevisiae* strain DY150, CEM‐GFP = human CD4+ T lymphoblastoid reporter cells, MAGIC‐5 = engineered to express CCR5 in HeLa‐CD4‐LTR/β‐Gal cells, MT‐4 = human T lymphoblast cells, BALB/c mice = Bagg Albino Laboratory‐bred; mice, THP‐1 = human acute monocytic leukemia; peripheral blood monocyte cells, Tg05 HBV transgenic mice = C57BL/6; 1.3mer overlength HBV genomic DNA, HL‐7702 = human hepatocytes cells, HepG2 = human liver carcinoma; hepatocellular cells, CNE1 = human nasopharyngeal carcinoma cells, C666‐1 = human nasopharyngeal carcinoma cells, NP361hTert = telomerase‐immortalized nonmalignant human nasopharyngeal epithelial cells, NP460hTert = telomerase‐immortalized nonmalignant human nasopharyngeal epithelial cells, NP550hTert = telomerase‐immortalized nonmalignant human nasopharyngeal epithelial cells, NP69 = human immortalized nasopharyngeal carcinoma cells, NP460 = human nasopharyngeal carcinoma cells, HaCaT = human immortalized keratinocytes cells, C33‐A = human uterus; cervix carcinoma epithelial cells, HEK293 = human embryonic kidney cells. Experimental intervention: CCCP = Carbonyl cyanide *m*‐chlorophenylhydrazone, TTFA = Thenoyltrifluoroacetone.

#### 2.2.1 RNA Viruses

##### 2.2.1.1 Hepatitis C Virus (HCV)

Hepatitis C virus (HCV), an enveloped positive‐sense single‐stranded RNA virus of the Flaviviridae family, replicates in the cytoplasm within membranous web structures derived from the endoplasmic reticulum, where viral RNA synthesis occurs. Hepatoma‐derived Huh7 cells support HCV RNA replication and are commonly used to study the viral life cycle. HCV infection induces host immune responses, including CD8^+^ T cell responses, although these immune cells are primarily used to investigate antiviral immunity rather than as systems for viral replication [81, 82, 83]. In HCV‐infected cells, early disruption of mitochondrial Ca^2^^+^ homeostasis reduces complex I activity and induces mitochondrial depolarization and increased ROS production [84]. The HCV core protein increases complex I‐dependent ROS production, and this increase is abolished by rotenone. Calcium addition to isolated mitochondria increases ROS production, which is further elevated by HCV core, whereas calcium chelation suppresses this effect [85]. Pharmacological interventions restoring complex I function, such as RU360 or amantadine, prevent Ca^2^^+^ overload, normalize membrane potential, and suppress reactive oxygen/nitrogen species (ROS/RNS) production. These treatments restore complex V activity and preserve cellular respiration [86, 87]. In addition, interaction analyses show that HCV proteins such as p7 and NS4B interact with mitochondrial complex V subunits, including ATP5H and ATP6 [88]. Gene set enrichment analyses of HCV‐specific CD8^+^ T cells show increased expression of OXPHOS and electron transport‐related genes during early infection, whereas these pathways decrease during chronic infection together with genes involved in mitochondrial metabolism and TCR signaling [89]. Transcriptomic profiling shows that during the later phase of HCV infection, expression of multiple mitochondrial respiratory chain subunits from complex I and complex IV is reduced, while ER stress‐related genes and pentose phosphate pathway enzymes are induced. These changes include decreased glycolysis and tricarboxylic acid (TCA) cycle intermediates, indicating virus‐induced metabolic reprogramming toward a state favoring long‐term persistence [90]. During prolonged HCV protein expression, enzymatic assays detect inhibition of complex I and complex V activities, while complexes III and IV remain largely unaffected. Despite this inhibition, intracellular ATP levels increase compared with noninduced cells, and this increase is dependent on the available energy substrate in the culture medium [91]. HCV‐driven suppression of key OXPHOS complexes occurs alongside shifts toward alternative ATP‐generating pathways. HCV shows distinct patterns of mitochondrial OXPHOS regulation across infection stages and experimental models, with increased OXPHOS‐related activity reported during early infection and suppression of specific respiratory complexes during prolonged infection.

##### 2.2.1.2 Zika Virus (ZIKV)

In Zika virus (ZIKV) infection, cytoplasmic replication is established within endoplasmic reticulum‐derived replication compartments, where viral RNA synthesis occurs, and virion assembly is linked to remodeled ER membranes [92, 93]. During ZIKV infection, mitochondrial metabolism undergoes stage‐ and cell type‐dependent regulation. In the early phase of infection, complex II activity supports both the TCA cycle and the ETC, driving OXPHOS to supply ATP required for viral replication. Inhibition of SDH by endogenous itaconate or by the pharmacological inhibitor lonidamine results in succinate accumulation and reduces ZIKV replication [94]. During infection, particularly in neural tissues, multi‐omics analyses identify downregulation of the TCA cycle and OXPHOS (including complex I, II, and IV components) at both the transcript and protein levels. These reductions are prominent in neurons and neuroblasts and are associated with mitochondrial dysfunction, neuronal cell death, and the neuropathology of ZIKV‐induced microcephaly [95]. These studies identify distinct patterns of OXPHOS regulation: stages with increased OXPHOS activity during early infection to promote viral replication, yet suppressed in later stages, correlating with host cell damage and disease progression. These stage‐ and cell type‐dependent differences in OXPHOS regulation should be considered when developing metabolism‐targeted antiviral strategies.

##### 2.2.1.3 West Nile Virus (WNV)

In West Nile virus (WNV) infection, a significant subset of mitochondria‐associated genes is suppressed, reflecting respiratory chain dysfunction and increased free radical production; similar transcriptional pathways are altered in neurodegenerative diseases such as Huntington's, Parkinson's, and Friedreich's ataxia. Similar neurological symptoms observed in WNV‐infected patients suggest shared pathogenic mechanisms [96, 97]. Reduced expression of genes involved in mitochondrial energy metabolism, including complex II subunit (SDHC), complex IV subunits (COX5B, COX6B), and multiple complex V components (ATP5G1, ATP5C1, ATP5J, ATP5B, ATP5F1A, ATP5O, ATP5F1), indicates decreased mitochondrial ATP generation, a known driver of neurodegeneration [98]. In a WNV mouse model that reproduces key features of WNV infection, RNA‐seq analysis of CNS tissue identified downregulation of multiple energy production genes, including COX6B2, a subunit of complex IV. This reduction in OXPHOS‐related gene expression occurred together with increased expression of genes involved in glycolytic metabolism. Pharmacological inhibition of PDK4, a negative regulator of PDH, with dichloroacetate (DCA) reduced infectious WNV titers. However, the functional contribution of the observed complex IV alteration was not directly examined [99].

##### 2.2.1.4 Severe Acute Respiratory Syndrome Coronavirus 2 (SARS‐CoV‐2)

For severe acute respiratory syndrome coronavirus 2 (SARS‐CoV‐2), viral RNA synthesis occurs within cytoplasmic double membrane vesicles generated from host intracellular membranes, which serve as sites of viral RNA replication. Multiple experimental systems, including primary human airway epithelial cells, lung tissues, and Vero E6 cells, have been used to investigate SARS‐CoV‐2 infection and host responses [100, 101]. During the early phase of SARS‐CoV‐2 infection, a transient increase in mitochondrial OXPHOS increases ATP production and supports viral replication [102]. Inhibition of succinate oxidation using dimethyl malonate reduces viral replication, indicating a proviral role for complex II at this stage [103]. In nasopharyngeal, lung, BALF, and NHBE samples, transcripts encoding mitochondrial respiration and OXPHOS components, particularly complex I subunits such as NDUFS2, NDUFB7, NDUFS6, NDUFAF6, NDUFB9, and NDUFV2, are reduced during SARS‐CoV‐2 infection. SARS‐CoV‐2 proteins target several aspects of mitochondrial function, ranging from mitochondrial protein synthesis and processing to OXPHOS and protein import. M and N proteins interact with components involved in mitochondrial protein synthesis and processing, including mitochondrial aminoacyl‐tRNA synthetases, COQ8B, and mitochondrial processing peptidases, whereas NSP6 and ORF9b associate with complex V subunits and TOMM70, respectively. These interactions indicate that SARS‐CoV‐2 can modulate mitochondrial function through protein‐specific mechanisms in addition to global transcriptional reprogramming. SARS‐CoV‐2 infection is also associated with increased mtROS and activation of HIF‐1α‐dependent glycolytic signaling, providing an indirect redox‐dependent mechanism linking impaired OXPHOS to metabolic reprogramming [104, 105]. ORF9c interacts with mitochondrial proteins NDUFAF1 and NDUFB9, potentially driving complex I suppression, while ORF3c increases succinate and ROS via reverse electron transport without inducing OXPHOS transcripts [106, 107]. Effector CD8^+^ T cells in COVID‐19 patients show downregulation of OXPHOS‐ and TCA cycle‐related genes, together with increased HIF‐1α signaling and glycolytic metabolism under hypoxic conditions [108]. Analysis of the OXPHOS/glycolysis ratio in cytotoxic T cells reveals that mild cases show a gradual OXPHOS increase over time, whereas severe cases exhibit high early OXPHOS that decreases during the mid‐infection stage, demonstrating disease severity‐associated differences in metabolic profiles [109]. Among myeloid cell subsets, airway A3 cells show elevated OXPHOS activity, positive correlation with TNF signaling and classical complement pathways, and a negative correlation with apoptosis, indicating a metabolically active, pro‐inflammatory phenotype [110]. Genomic and proteomic analyses show that COVID‐19 patients harbor mtDNA mutations in complex V subunit (ATP6) and complex III subunit (CYTB), as well as pathogenic variants in complex I subunits (ND4, ND4L, ND5, ND2) and complex IV subunit (COX1), leading to reduced enzymatic efficiency, impaired ATP synthesis, and altered ROS production [111, 112]. In Vero E6 cells, OXPHOS transcripts encoded by the mitochondrial genome increase while those encoded by the nuclear genome decrease, accompanied by ATP depletion. Replacing glucose with galactose reduces viral replication, highlighting crosstalk between OXPHOS and glycolysis [113]. Proteomic analysis of hepatic tissues from fatal cases shows global OXPHOS and respiration suppression alongside heightened interferon responses [114]. In A549 and HBE cells expressing Ad5‐N, mtROS levels rise with upregulated mitochondrially encoded complex I and III subunits; despite increased complex I and pronounced complex III activity, ATP production declines, indicating inefficient electron transport coupling. Mechanistically, the SARS‐CoV‐2 N protein associates with the mitochondrial transcription components POLRMT, TFB2M, and TFAM and stabilizes these proteins by limiting their proteasome‐dependent degradation. This promotes the transcription of mitochondrially encoded complex I and III subunits and is accompanied by increased mtROS and activation of the stress‐responsive JNK and p38 pathways [115].

##### 2.2.1.5 Influenza Virus

A defining feature of influenza viruses, segmented negative‐sense RNA viruses of the Orthomyxoviridae family, is that transcription and replication of viral RNA occur in the host cell nucleus before nuclear export and subsequent virion assembly [116]. Madin–Darby canine kidney (MDCK) cells and human lung epithelial A549 cells are widely used in vitro systems for studying influenza virus replication [117]. Studies on influenza virus host interactions demonstrate that OXPHOS regulation during infection differs across viral subtypes, host cell types, and tissues. In H5N1‐infected MDCK cells, COX4I1, a nuclear‐encoded subunit of complex IV, is upregulated, and its overexpression enhances viral replication, whereas small interfering (siRNA) mediated silencing reduces viral yield and impairs viral ribonucleoprotein (vRNP) nuclear export. Lycorine, an inhibitor of vRNP export, further suppresses COX4I1 expression without cytotoxicity; time of addition studies confirm its inhibitory effect across multiple replication cycles [118, 119]. In contrast, H1N1‐infected human lung epithelial A549 cells exhibit miR4276‐mediated downregulation of COX6C, with early upregulation and later suppression of COX6C correlating with viral replication dynamics [120]. At the tissue level, transcriptomic analysis of subcutaneous adipose tissue (SCAT) from H3N2‐infected mice shows reduced expression of genes involved in OXPHOS, the TCA cycle, and glycolysis, with downregulation of complex I genes [121]. The functional effects of individual OXPHOS components differ among influenza virus models. COX4I1 upregulation supports H5N1 replication in MDCK cells, whereas COX6C suppression promotes H1N1 replication in A549 cells. In contrast, H3N2 infection is associated with OXPHOS downregulation in SCAT. These results demonstrate distinct patterns of OXPHOS regulation across viral subtypes and experimental models.

##### 2.2.1.6 Rabies Virus (RABV)

Rabies virus (RABV), a negative‐sense single‐stranded RNA virus of the Rhabdoviridae family, replicates in the cytoplasm of infected cells within Negri bodies, which function as sites for viral transcription and replication [122]. RABV infection increases respiratory chain complex I activity in dorsal root ganglion neurons, PC12 cells, and MNA cells, with greater increases observed in cell types with higher susceptibility to infection. Complex IV activity is also elevated in all cell types but shows a weaker association with susceptibility, whereas complex II and III activities remain largely unchanged [123]. In HEK‐293T cells expressing individual RABV proteins, the phosphoprotein (P) increases complex I activity and ROS production, whereas the M protein reduces complex I activity. In contrast, N and G proteins have no measurable effects on complex I activity. Mitochondrial localization and co‐immunoprecipitation with complex I further characterize the mitochondrial association of P. Deletion mapping identifies aa139‐172 as the functional region required for P‐mediated increases in complex I activity and ROS production [124]. In human RABV‐infected brain regions, transcriptomic profiling reveals upregulation of multiple subunits from complexes I, III, IV, and V, with significant activity increases for complexes I, IV, and V, whereas complex III activity does not significantly change. In canine brains, complexes I and IV are elevated in both frontal cortex and cerebellum, complex II increases only in frontal cortex, and complex V remains largely unchanged. In both species, OXPHOS activity changes do not correlate with viral load, suggesting that these alterations arise from mitochondrial responses to infection rather than direct effects of viral replication [125].

##### 2.2.1.7 Mokola Virus

Mokola virus matrix protein (M‐MOK) directly associates with complex IV subunit 1 (COX1) in the mitochondrial respiratory chain, resulting in reduced enzyme activity and induction of apoptosis. Mapping of M‐MOK identified a mitochondrial targeting region spanning aa46‐110, within which an α‐helical region at aa69‐82 contributes to mitochondrial targeting and functional disruption. K77 and N81 within this region are critical functional residues, as their substitution restores COX activity and abolishes M‐MOK‐induced cytochrome c release and apoptosis. In addition, the COX1 aa104‐125 region was identified as an M‐MOK interacting region, further defining the molecular basis of the M‐MOK‐COX1 interaction [126].

##### 2.2.1.8 Human Immunodeficiency Virus (HIV)

Following entry, human immunodeficiency virus type 1 (HIV‐1), a retrovirus of the Retroviridae family, reverse‐transcribes its RNA genome into DNA, integrates the resulting viral DNA into the host genome as a provirus, and subsequently assembles at the plasma membrane [127, 128]. HIV infection alters OXPHOS across multiple cell types and tissues, with respiratory chain complexes showing both increased and decreased activities. In peripheral blood mononuclear cells (PBMCs) from patients with HIV, mtDNA copy number and the activities of complexes III and IV are reduced, together with decreased activity of nuclear‐encoded complex II [129]. Reduced mitochondrial protein expression is also observed in HIV associated sensory neuropathy and SIV‐induced neuropathy, including reduced COX1 expression in peripheral nerve tissue [130]. At the subunit level, HIV‐1 NL4.3 infection selectively downregulates the complex I subunit (NDUFA6) in CEM‐GFP cells, PBMCs, and Jurkat cells, leading to a threefold decrease in rotenone‐sensitive complex I activity, while azidothymidine (AZT) treatment attenuates this effect [131]. In HIV‐1‐infected CEM‐GFP T cells, complex IV activity is increased in a multiplicity‐of‐infection‐dependent manner, whereas complexes I, II, and III show significant reductions and complex V is modestly elevated [132]. Proteomic profiling of CD4^+^ T cell subsets from long‐term nonprogressors reveals a distinct upregulation of OXPHOS pathways accompanied by downregulation of glycolysis, suggesting a metabolic shift toward mitochondrial respiration [133]. Functional assays demonstrate that under glucose‐restricted conditions, HIV‐1 replication required glutamine‐dependent OXPHOS, as inhibition of mitochondrial respiration by oligomycin (complex V inhibitor) or AMA (complex III inhibitor) completely blocks infection, whereas glutamine deprivation reduces infection independently of these inhibitors [134]. Inhibition of mitochondrial respiration with rotenone (complex I inhibitor) or AMA markedly reduces replication of VSV‐G pseudotyped HIV‐1 in Jurkat T cells, and metformin, a complex I‐targeting antidiabetic drug, suppresses OXPHOS and reduces viral titers in both Jurkat cells and primary CD4^+^ T cells infected with clinical isolates [135]. Vpr expression in yeast reduces the activities of complexes I, III, and IV as well as citrate synthase, while complex II activity is also decreased, indicating a broad inhibitory effect on mitochondrial enzymes [136]. Among HIV‐1 proteins, the p2 peptide enhances complex IV activity in a dose‐dependent manner. The p2 peptide interacts with the intermembrane space‐exposed N214‐F235 region of COX1, with Gln6 within the conserved AEAMSQ motif serving as a critical residue for this interaction. Substitution of Gln6 reduces p2‐dependent ATP production and late viral cDNA synthesis [137]. In contrast, the full‐length Tat protein strongly inhibits complex IV activity in mitochondria from multiple human and mouse tissues, an effect not observed with shorter Tat fragments or other viral peptides [138]. These findings show that HIV‐1 alters OXPHOS through both direct actions of viral proteins and broader metabolic reprogramming, with complex I inhibition and complex IV regulation observed across different experimental models.

##### 2.2.1.9 Dengue Virus (DENV)

Dengue virus (DENV), a positive‐sense single‐stranded RNA virus of the Flaviviridae family, encodes the nonstructural protein NS3, which has both protease and helicase activities essential for viral replication. DENV NS3 associates with mitochondria and selectively impairs respiratory complex I activity. The NS3 protease domain inhibits oxidoreductase activity in a concentration‐dependent manner, while complexes II–V remain unaffected. NS3 reduces complex I‐dependent respiration driven by malate/pyruvate without altering leak respiration or ΔΨm. The catalytically inactive NS3pro S135A mutant fails to significantly inhibit complex I, demonstrating NS3 protease activity is required for complex I inhibition. In DENV‐2‐infected Huh7 cells, complex I activity is also reduced, further supporting the selective suppression of complex I during DENV infection [139].

#### 2.2.2 DNA Viruses

##### 2.2.2.1 Hepatitis B Virus (HBV)

Hepatitis B virus (HBV) is a partially dsDNA virus of the Hepadnaviridae family that replicates through reverse transcription of pregenomic RNA within nucleocapsids, followed by virion assembly and secretion from hepatocytes. Both clinical samples from chronic hepatitis B (CHB) patients and hepatocyte‐derived in vitro systems, including Huh7 cells, have been used to investigate HBV‐associated metabolic alterations [140, 141, 142, 143]. In CHB‐infected patients, mitochondrial OXPHOS enzyme activities are altered in a complex, specific manner. In untreated CHB patients, nuclear DNA‐encoded enzyme activities, including complex II, are markedly reduced compared with healthy controls, along with lower carnitine levels, whereas mtDNA‐encoded enzyme activities remain comparable between treated and untreated groups [144]. In hepatocyte‐derived HL‐7702 cells expressing HBx, the mitochondrial complex IV subunit (COX3) protein is upregulated without a corresponding increase in mRNA, indicating posttranscriptional regulation. HBx also associates with COX3, with HBx aa72‐117 required for its interaction with COX3. COX activity also increases, COX activity is significantly elevated, whereas ΔΨm remains unchanged [145, 146]. In a separate study, enzymatic assays show that HBx reduces the activities of complexes I, III, IV, and V, while complex II activity remains unchanged [147]. Transcriptomic analysis of THP‐1 macrophages demonstrates that HBV infection upregulates OXPHOS activity, driving metabolic reprogramming that suppresses IL‐1β production, an antiviral cytokine. Pharmacological inhibition of OXPHOS with dimethyl malonate restores IL‐1β expression specifically in HBV‐infected cells. Exogenous IL‐1β treatment in Huh7 cells and HBV transgenic mice reduces viral replication, whereas IL‐1β neutralization reverses this antiviral effect [148]. These observations indicate that HBV modulates OXPHOS differently across cell types and experimental conditions, enhancing activity in some settings to suppress antiviral responses and promote persistence, while selectively reducing respiratory complex activity under other conditions.

##### 2.2.2.2 Epstein–Barr Virus (EBV)

After primary infection, Epstein–Barr virus (EBV), a dsDNA virus of the Herpesviridae family, establishes latent infection in B cells and, in latent membrane protein 1 (LMP1) expressing latency programs, promotes cellular transformation and proliferation through latent viral signaling [149, 150]. Primary human B cells and nasopharyngeal epithelial cell models are widely used to study EBV‐induced metabolic reprogramming. In primary human B cells undergoing de novo EBV infection, proteomic profiling across multiple time points reveals enhanced mitochondrial one‐carbon metabolism and upregulation of ETC components from both nuclear and mitochondrial genomes, together with increased basal and maximal OCR. This oxidative capacity is essential for B cell outgrowth, as treatment with piericidin A (complex I inhibitor) or AMA (complex III inhibitor) suppresses proliferation [151]. Overexpression of the viral LMP1 induces DNA methyltransferase 1 (DNMT1) translocation into mitochondria, where DNMT1 increases mtDNA methylation and reduces expression of OXPHOS genes such as complex I subunit (ND6), complex V subunit (ATP6), and complex IV subunit (COX2). LMP1 depletion or inhibition of DNMT1 with 5‐aza‐2′‐deoxycytidine restores expression of these genes [152]. EBV‐infected nasopharyngeal epithelial cells show a metabolic shift with increased glycolytic enzymes, reduced OXPHOS protein levels, elevated extracellular acidification rate (ECAR), and diminished OCR. These cells are more sensitive to 2‐deoxy‐d‐glucose than controls, while uninfected cells are more affected by oligomycin, indicating greater dependence on glycolytic metabolism in infected cells. Suppression of LMP1 restores OXPHOS activity and limits lactate production, whereas LMP1 expression shifts cellular metabolism toward glycolysis with diminished oxygen consumption [153].

##### 2.2.2.3 Human Papillomavirus (HPV)

Completion of the human papillomavirus (HPV) life cycle depends on epithelial differentiation, as this small dsDNA virus of the Papillomaviridae family couples productive replication to the maturation of infected epithelial cells [154, 155, 156]. HPV E2 interacts with mitochondrial inner membrane proteins, many linked to OXPHOS, particularly complex III. Its expression elevates mitochondrial superoxide detected by MitoSOX, indicating disruption of OXPHOS activity [157]. HPV E7 protein associates with ATP5B, a subunit of mitochondrial complex V. Substitution of L23 in HPV8 E7 weakens this association, identifying L23 as an important determinant of the E7‐ATP5B interaction. E7 also co‐localizes with cytochrome c. These findings support a role for HPV proteins in altering mitochondrial oxidative metabolism through association with key respiratory components [158].

Together, these studies show that OXPHOS is differentially regulated during viral infection, with some alterations functionally linked to viral genome replication, viral protein expression, or infectious virion production, and others observed without direct functional validation of their effects on viral infection outcomes. Accordingly, Table 1 summarizes OXPHOS alterations functionally linked to viral infection outcomes, whereas Table 2 summarizes infection‐associated OXPHOS alterations for which effects on viral infection outcomes have not been directly established.

The OXPHOS alterations described in this section and the complementary mitochondrial processes examined in Section 3 together provide a broader view of how individual viruses modulate mitochondrial function. For RSV, the discussion extends to mitochondrial positioning, membrane potential, and mtROS signaling; for VACV, to mitochondrial fatty acid utilization; and for DENV, to substrate metabolism, mitochondrial dynamics, and MAVS‐dependent signaling. SARS‐CoV‐2 and HIV are further examined in relation to their respective effects on cellular metabolism, redox regulation, and mitochondrial membrane properties, structure, or dynamics. The discussion of HCV expands to calcium homeostasis, membrane potential, mitochondrial dynamics, and mitophagy, while that of ZIKV addresses respiratory and glycolytic capacities and membrane potential. For influenza virus, the focus shifts to PB1‐F2‐mediated depolarization and apoptosis, and for HBV, to calcium signaling, membrane potential, and mitochondrial genomic alterations.

## 3 Mitochondrial Function Beyond OXPHOS

Beyond OXPHOS complexes, viral infection alters diverse mitochondrial processes, including mitochondrial metabolism, dynamics, membrane potential, redox homeostasis, and signaling. These changes can influence viral infection as well as mitochondrial integrity and cellular responses. Increased ROS production and loss of mitochondrial ΔΨm are observed across multiple viral infections, as illustrated in Figure 2. Section 2 distinguishes mitochondrial alterations functionally linked to viral infection outcomes from those observed during infection without direct functional validation of their effects on these outcomes.

**FIGURE 2:**
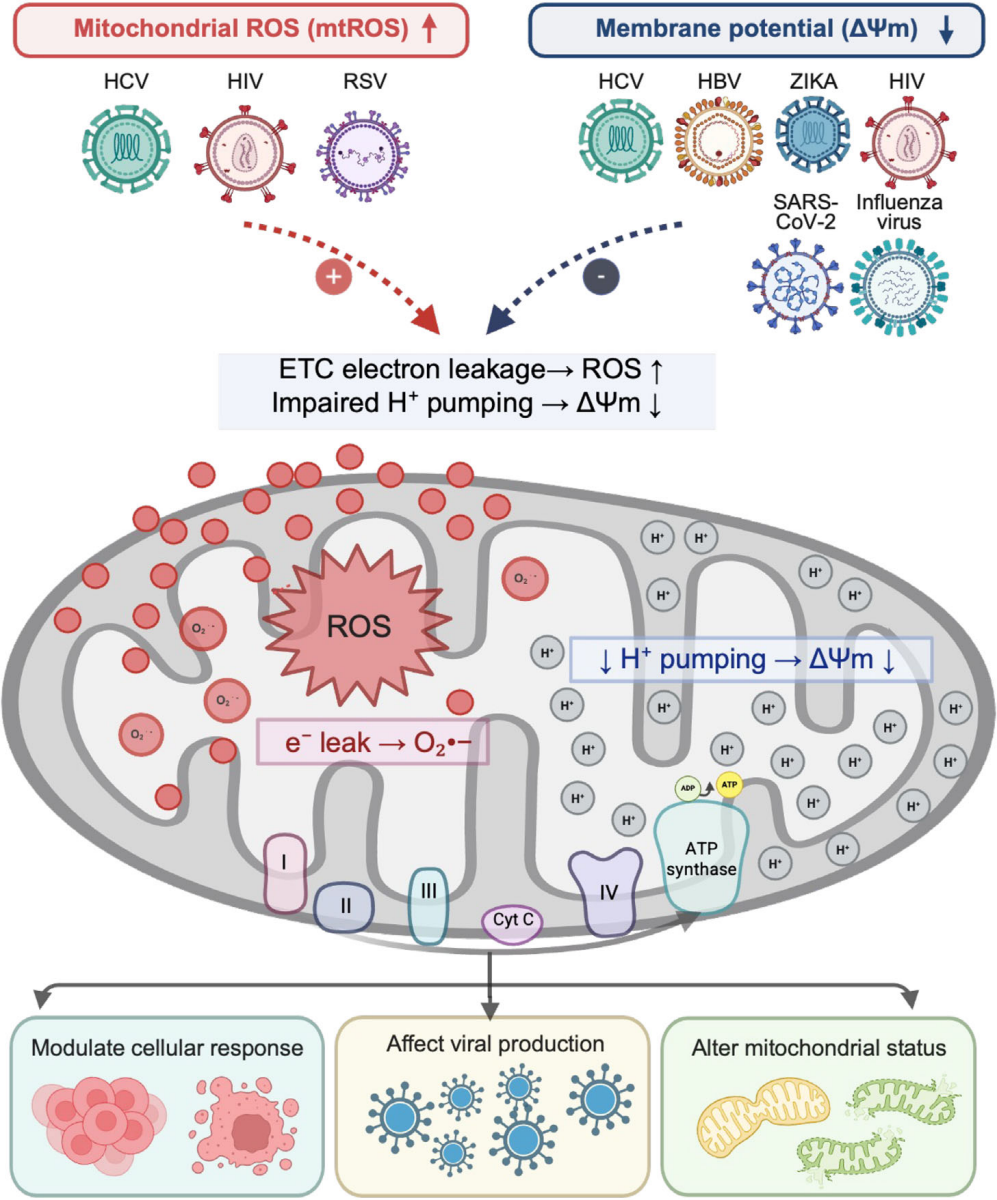
Virus‐induced alterations in mitochondrial membrane potential (ΔΨm) and ROS production. Viral infections can alter mitochondrial redox homeostasis and membrane potential through perturbation of mitochondrial function. Disruption of electron transport can promote electron leakage and increase reactive oxygen species (ROS) production, whereas impaired proton pumping across the inner mitochondrial membrane reduces the electrochemical gradient required to maintain mitochondrial ΔΨm, leading to mitochondrial depolarization. Increased mtROS production has been reported during HCV, HIV, and RSV infection, whereas loss of ΔΨm has been observed during HCV, HBV, ZIKV, HIV, SARS‐CoV‐2, and influenza virus infection. These mitochondrial alterations can affect viral replication and production, alter mitochondrial integrity and dynamics, and modulate cellular responses, including cell survival, apoptosis, and immune signaling.

### 3.1 Mitochondrial Determinants of Viral Infection

Mitochondrial processes beyond the OXPHOS complexes have diverse effects on viral infection, either promoting or restricting infection. As summarized in Table 3, functional studies have linked alterations in mitochondrial metabolism, morphology and dynamics, redox regulation, membrane potential, and mitochondrial integrity to viral infection outcomes. Mitochondrial functions beyond OXPHOS can influence different stages and outcomes of viral infection through metabolic reprogramming, mitochondrial remodeling, redox regulation, and changes in mitochondrial homeostasis. The following subsections examine the molecular mechanisms underlying these effects across different viral infections.

**TABLE 3:** Mitochondrial alterations functionally linked to viral infection.

Virus Genome	Virus	Effect on Viral Infection	Viral protein	Mitochondrial Target/Process	Experimental Model	Experimental Intervention	Functional Outcome	References
RNA	SARS‐CoV‐2	Proviral		DRP1, CypD	Vero E6, Huh‐7 cell lines	Mdivi‐1 (5 µM), CsA (40 µM)	MPTP opening supports SARS‐CoV‐2 replication	[160]
				Glycolysis, OCR	Patient PBMC samples		Metabolic reprogramming associates with COVID‐19 severity	[161]
	RSV	Proviral		ROS	Patient blood samples A549 cell line, BALB/c mice	MitoQ (500 µM)	mtROS facilitates infectious RSV production	[162]
	HIV	Proviral		Mitochondrial enzyme activity	ACH2, A3.01 cell lines		Mitochondrial HIV RNA contributes to cytopathogenicity	[163]
				Glycolysis	Patient urethral tissue samples		Glycolysis supports HIV‐1 latency reversal	[164]
			Tat	OCR	Patient blood samples THP‐1, BEAS‐2B cell lines		HIV‐associated exosomes impair mitochondrial bioenergetics	[165]
			Vpr	ΔΨm	HEK293 cell line	shRNA (Mfn2, DDB1, VprBP, CUL4A)	Vpr‐mediated Mfn2 loss disrupts mitochondrial integrity	[166]
			Tat	ΔΨm, ROS	K562 cell line		Tat sensitizes cells to mitochondrial apoptosis	[167]
			Vpr	ΔΨm	Patient PBL samples Jurkat cell line		Vpr‐induced MPTP opening triggers apoptosis	[168]
	DENV	Proviral		CPT1	Huh‐7 cell line	Etomoxir (400 µM), AICAR (4 mM)	Mitochondrial β‐oxidation promotes DENV replication	[169]
			NS4B	DRP1	Huh‐7 cell line	shRNA (DRP1)	NS4B inhibits DRP1, promoting DENV replication	[170]
DNA	VACV	Proviral		CPT1	BSC40 cell line	Etomoxir (360 µM)	Fatty‐acid oxidation supports VACV virion production	[171]

Abbreviations: Virus: SARS‐CoV‐2 = severe acute respiratory syndrome coronavirus 2, RSV = respiratory syncytial virus, HIV = human immunodeficiency virus, DENV = Dengue virus, VACV = Vaccinia virus. Viral protein: ORF9c = open reading frame 9c, Tat = trans‐activator of transcription, Vpr = viral protein R. Mitochondrial target/process: DRP1 = dynamin‐related protein‐1, CypD = cyclophilin D, OCR = oxygen consumption rate, ROS = reactive oxygen species, ΔΨm = mitochondrial membrane potential, CPT1 = Carnitine palmitoyltransferase I. Experimental Model: Vero E6 = African green monkey kidney epithelial cells, Huh‐7 = human hepatoma‐derived carcinoma cells, PBMC = peripheral blood mononuclear cells, A549 = alveolar epithelial cells; human lung carcinoma epithelial cells, BALB/c mice = Bagg Albino Laboratory‐bred; mice, ACH2 = uninfected parental A3.01‐derived T‐lymphocytic cells; model for chronic HIV‐1 infection, possesses a single integrated copy of the HIV‐1 strain LAI, A3.01 = human precursor T‐cell acute lymphoblastic leukemia; CD4+ T cells; derived from a Jurkat cells, THP‐1 = human acute monocytic leukemia; peripheral blood monocyte cells, BEAS‐2B = human lung; bronchus epithelial cells, hµglia = human microglia cells, PBLs = peripheral blood lymphocytes, Jurkat = human CD4+ T lymphoblast cells, HEK293 = human embryonic kidney cells, K562 = human bone; marrow lymphoblast cells, BSC40 = African green monkey kidney epithelial cells. Experimental intervention: Mdivi‐1 = mitochondrial division inhibitor 1, CsA = Cyclosporin A, MitoQ = mitoquinone mesylate, AICAR = 5‐aminoimidazole‐4‐carboxamide ribonucleotide.

#### 3.1.1 RNA Viruses

##### 3.1.1.1 SARS‐CoV‐2

SARS‐CoV‐2 targets host mitochondria through direct protein–protein interactions and by altering mitochondrial bioenergetics. Proteomic mapping identifies viral proteins such as NSP4, NSP8, and ORF9c that associate with mitochondrial host proteins and affect mitochondrial pathways involved in viral replication [159]. In Vero E6 and Huh‐7 cells, SARS‐CoV‐2 infection induces progressive loss of ΔΨm, mitochondrial permeability transition pore (MPTP) opening, increased ROS production, and mitochondrial structural alterations, including swelling and vacuolization. Viral double‐stranded RNA (dsRNA) accumulates within mitochondria, and this localization is facilitated by the mitochondrial outer membrane receptor Tom20. Tom20 silencing reduces mitochondrial dsRNA localization and mitochondrial N protein levels, and reduces ROS production and viral replication, supporting a functional role for Tom20 in SARS‐CoV‐2‐induced mitochondrial dysfunction. Pharmacological stabilization of mitochondrial function with mitochondrial division inhibitor 1 (Mdivi‐1) or inhibition of MPTP opening with cyclosporin A (CsA) preserves mitochondrial integrity and significantly reduces viral load, indicating that ΔΨm loss and MPTP opening contribute to efficient viral replication [160]. In PBMCs from COVID‐19 patients, ATP‐linked respiration, reserve capacity, and maximal respiration are reduced, reflecting impaired mitochondrial function. Basal and stressed ECAR are also elevated, with greater glycolytic activity observed in these cells [161]. These findings show that SARS‐CoV‐2 alters ΔΨm, permeability, structure, and bioenergetics, while pharmacological modulation of mitochondrial dysfunction reduces viral replication.

##### 3.1.1.2 RSV

RSV‐infected cells exhibit a progressive loss of ΔΨm at 16–18 hpi, with increased mtROS production that contributes to RSV replication. Treatment with the mtROS scavenger MitoQ reduces RSV genome copies and infectious titers, and restores mitochondrial morphology and distribution in both A549 cells and primary human bronchial epithelial cells (pBECs). In a mouse model, MitoQ treatment significantly lowers viral replication and reduces lung and systemic inflammation, demonstrating that mtROS contributes to RSV replication and inflammatory responses [162].

##### 3.1.1.3 HIV

HIV infection alters mitochondrial function beyond direct effects on OXPHOS complexes. In T cells, viral RNA rapidly accumulates in mitochondria within the first hour of induction, progressively impairs mitochondrial enzymatic activity while cell viability is initially maintained, indicating that RNA trafficking itself destabilizes organelle function [163]. In macrophages, S100A8 triggers latency reversal via glycolysis, and blocking glycolysis completely prevents reactivation, demonstrating the metabolic dependency of reservoirs on host energy pathways [164]. Viral proteins such as Tat and gp120 promote the expression of specific miRNAs, including miR‐27a, that are packaged into exosomes. Transfer of these vesicles to epithelial cells suppresses oxidative capacity, reduces oxygen consumption, and lowers ATP levels in recipient cells [165]. Vpr disrupts mitochondrial integrity, which promotes ubiquitin‐mediated degradation of Mfn2 through the VprBP–DDB1–CUL4A complex, causing ΔΨm dissipation and organelle destabilization [166]. Tat expression alone does not induce apoptosis but primes nutrient‐stressed cells to undergo ΔΨm loss followed by ROS accumulation, with mitochondrial depolarization promoting stress‐enhanced cell death [167]. Synthetic Vpr, particularly its C‐terminal fragment (Vpr52‐96), is sufficient to trigger early ΔΨm collapse and apoptosis in Jurkat and primary lymphocytes. Within this region, the Vpr71‐82 sequence containing the H(S/F)RIG motifs is sufficient to induce mitochondrial membrane permeabilization, whereas substitution of R73, R77, or R80 attenuates these mitochondrial effects. The c‐terminal region of Vpr52‐96 also binds purified adenine nucleotide translocator (ANT) and a molecular complex containing ANT and the voltage‐dependent anion channel (VDAC), both components of the mitochondrial permeability transition pore complex (PTPC) [168]. HIV‐induced changes in ΔΨm, mitochondrial dynamics, and metabolism affect cellular survival and processes associated with viral latency.

##### 3.1.1.4 DENV

DENV infection reprograms mitochondrial substrate utilization. In Huh7 cells, infection suppresses glutamine oxidation while increasing glucose metabolism. However, glucose primarily serves an anaplerotic function that facilitates the oxidation of endogenous fatty acids rather than acting directly as the major oxidative substrate. Etomoxir inhibits CPT1‐dependent mitochondrial fatty acid transport and suppresses DENV E protein expression, viral RNA accumulation, and production of infectious particles. In contrast, 5‐aminoimidazole‐4‐carboxamide ribonucleotide (AICAR), an adenosine monophosphate‐activated protein kinase (AMPK) activator that promotes mitochondrial fatty acid β‐oxidation through ACC inhibition, enhances DENV replication, further supporting a proviral role for mitochondrial fatty acid utilization [169]. In addition to metabolic reprogramming, DENV modifies mitochondrial dynamics. DENV NS4B associates with mitochondrial proteins and promotes mitochondrial elongation by reducing dynamin‐related protein‐1 (DRP1) phosphorylation at Ser616 and its recruitment to mitochondria. The 2K signal sequence of NS4B is required for this regulation, as membrane‐associated 2K‐NS4B decreases both CDK1 expression and DRP1 Ser616 phosphorylation, whereas NS4B lacking the 2K sequence fails to induce these effects. DRP1 depletion enhances mitochondrial elongation and DENV replication, whereas MFN2 depletion promotes mitochondrial fragmentation and suppresses viral replication. This mitochondrial remodeling is associated with disruption of MAMs, reduced RIG‐I recruitment to MAMs, and attenuation of MAVS‐dependent antiviral signaling and downstream interferon responses [170].

#### 3.1.2 DNA Viruses

##### 3.1.2.1 VACV

VACV triggers a pseudo‐hypoxic state early in infection, and blockade of fatty acid synthase with cerulenin suppresses viral replication, supporting a functional role for lipid metabolism in VACV infection. Import of long‐chain fatty acids into mitochondria via CPT1 is essential, as inhibition with etomoxir reduces viral yield. This effect becomes more pronounced under glucose‐free conditions. VACV replication depends predominantly on mitochondrial β‐oxidation and OXPHOS rather than glycolysis for energy production [171].

### 3.2 Mitochondrial Responses during Viral Infection

Table 4 summarizes alterations in mitochondrial processes other than OXPHOS complexes observed during viral infection whose functional consequences remain incompletely defined. Although these studies identify changes in mitochondrial metabolism, dynamics, membrane potential, redox regulation, or signaling, they do not establish a functional link between the specific mitochondrial alteration and the viral infection outcomes defined above. Therefore, these alterations are not classified as direct proviral or antiviral determinants but are interpreted as mitochondrial responses associated with viral infection. This section highlights mitochondrial responses whose functional significance in viral infection remains to be established.

**TABLE 4:** Mitochondrial alterations during viral infection.

Virus Genome	Virus	Viral protein	Mitochondrial target/process	Experimental model	Experimental intervention	Observed mitochondrial changes	References
RNA	HCV	Core	Hsp60	Huh‐7 cell line		HCV core–Hsp60 interaction increases ROS and reduces apoptosis	[175]
		p7	ΔΨm	C57BL/6N mice		HCV p7 decreases mitochondrial ΔΨm	[178]
		NS5A	ΔΨm	Huh‐7.5 cell line	CCCP (10 µM)	NS5A reduces ΔΨm and induces mitophagy	[179]
	ZIKV	M	ΔΨm, OCR	MRC5 cell line		ZIKV strains reduce ΔΨm and OXPHOS	[180]
	RV		OCR/ECAR	Vero, A549, HUVECs cell lines		RV infection increases mitochondrial metabolic activity	[181]
	Influenza virus	PB1‐F2	Cellular respiration	MDCK cell line		PB1‐F2 disrupts ΔΨm and induces apoptosis	[184]
DNA	HBV	HBx	ΔΨm, Cytochrome c	HepG2, Huh‐7 cell lines		HBx reduces ΔΨm and induces cytochrome c release	[185]
		HBx	ΔΨm	COS‐1, Huh‐7 cell lines		HBx decreases mitochondrial ΔΨm	[186]
		HBx	MPTP	HepG2 cell line	CsA (10 µM)	HBx alters mitochondrial Ca^2^^+^ signaling	[187]

Abbreviations: Virus: HCV = Hepatitis C virus, ZIKV = Zika virus, RV = Rubella virus, HBV = Hepatitis B Virus. Viral protein: p7 = protein 7, NS4B = nonstructural protein 4B, Core = Capsid protein, M = membrane protein, PB1‐F2 = polymerase basic protein 1 ‐ Frame 2, HBx = HBV X protein. Mitochondrial target/process: Hsp60 = heat shock protein 60, ΔΨm = mitochondrial membrane potential, OCR = oxygen consumption rate, ECAR = extracellular acidification rate, MPTP = mitochondrial permeability transition pore. Experimental Model: C57BL/6N = C57 black 6 NIH mice, Huh‐7.5 = human hepatoma‐derived carcinoma cells, Huh‐7 = human hepatoma‐derived carcinoma cells, MRC5 = Medical Research Council cell strain 5; human fetal lung fibroblast cells, Vero = African green monkey kidney epithelial cells, A549 = alveolar epithelial cells; human lung carcinoma epithelial cells, HUVECs = human umbilical vein endothelial cells, MDCK = Madin–Darby canine kidney; cocker spaniel; dog kidney epithelial cells, HepG2 = human liver carcinoma; hepatocellular cells, experimental intervention: CCCP = carbonyl cyanide *m*‐chlorophenylhydrazone, CsA = cyclosporin A.

#### 3.2.1 RNA Viruses

##### 3.2.1.1 HCV

HCV infection alters mitochondrial function through multiple viral proteins. The core protein perturbs the ETC through its interaction with Hsp60, leading to excessive ROS generation and increased susceptibility to apoptosis [172, 173, 174, 175]. ΔΨm is also affected by HCV proteins, with p7 decreasing ΔΨm in a dose‐dependent manner and HCV infection of hepatoma cells producing a similar decrease [84, 176, 177, 178]. Mitochondrial integrity is further disrupted by NS5A through decreased ΔΨm and altered mitochondrial dynamics, which promote mitophagy. Inhibition of autophagosome–lysosome fusion further enhances this effect, while p62 degradation is also observed during autophagy [177, 179]. HCV proteins alter mitochondrial homeostasis through increased ROS production, loss of ΔΨm, and changes in mitochondrial dynamics [175, 178, 179].

##### 3.2.1.2 ZIKV

OXPHOS is disrupted during ZIKV infection. Seahorse metabolic profiling shows that both glycolytic and respiratory capacities decline after infection with either the wild‐type or the M‐F37L variant, and these reductions occur independently of viral load or overall cell survival. Analyses of ΔΨm further indicate that pathogenic ZIKV strains are associated with diminished mitochondrial activity and impaired OXPHOS [180].

##### 3.2.1.3 RV

Infection with the Therian and Wb‐12 strains increases the OCR/ECAR ratio, indicating enhanced mitochondrial metabolic activity, despite lower replication of Wb‐12 compared with Therien in Vero cells. Similar elevations in mitochondrial function are also observed in Wb‐12‐infected A549 cells and HUVECs. These changes support a shift toward oxidative metabolism during infection [181].

##### 3.2.1.4 Influenza Virus

The influenza A virus protein PB1‐F2 associates with the mitochondrial inner membrane, leading to loss of ΔΨm and induction of apoptosis. At the molecular level, PB1‐F2 targets mitochondria through a C‐terminal mitochondrial targeting sequence, with the aa69‐82 region forming an amphipathic α‐helix that mediates its association with the inner mitochondrial membrane. An overlapping region spanning aa63‐75 is required for efficient mitochondrial localization and induction of apoptosis, with the basic residues Lys73 and Arg75 serving as critical determinants of mitochondrial targeting. In addition to these PB1‐F2‐mediated mitochondrial effects, influenza infection progressively reduces mitochondrial respiration. Oxygen consumption decreases by approximately 30% at 6 hpi and 45% at 12 hpi [182, 183]. Cellular respiration is progressively suppressed, with greater reductions observed as infection progresses [184].

#### 3.2.2 DNA Viruses

##### 3.2.2.1 HBV

HBV infection alters mitochondrial homeostasis through multiple HBx‐associated processes. HBx localizes to mitochondria, where it reduces ΔΨm, promotes cytochrome c release, and disrupts respiratory activity. These mitochondrial effects are observed in liver‐derived cells, and HBx also co‐localizes with mitochondrial proteins such as COX1 [24, 185, 186]. HBx expression elevates cytosolic Ca^2^^+^ levels during viral replication and partially overlaps with mitochondrial compartments. CsA inhibits this HBx‐induced Ca^2^^+^ increase, supporting a role for HBx in modulating calcium flux and mitochondrial signaling [187]. Studies using Orai1 siRNA show that blockade of store‐operated calcium entry (SOCE) reduces both Ca^2^^+^ influx and HBV replication, demonstrating that Orai1‐dependent Ca^2^^+^ entry supports HBV replication [188]. At the genomic level, HBV DNA integrations are frequently detected in mitochondrial genes encoding OXPHOS subunits. These genes include complex I subunits (ND1, ND2, ND4, ND5), complex III subunit CYTB, complex IV subunit COX1, and complex V subunit ATP6. In patient tumor tissues, these integration events correlate with higher viral DNA and pregenome RNA levels, suggesting that HBV integration into mtDNA perturbs OXPHOS and supports viral replication [189]. HBV infection remodels mitochondrial function through changes in ΔΨm, calcium homeostasis, protein–protein interactions, and mitochondrial genomic alterations. Pharmacological inhibition with CsA and genetic interventions targeting SOCE show that mitochondrial activity is associated with the regulation of viral replication [24, 187, 188, 189].

Beyond direct effects on OXPHOS complexes, diverse mitochondrial functions are altered during viral infection through both virus‐mediated regulation and infection‐associated cellular changes. Several mitochondrial processes, including membrane potential regulation, ROS signaling, and mitochondrial dynamics, have demonstrated effects on viral replication and replication‐associated processes (Table 3), whereas other mitochondrial alterations occur without direct functional validation of their effects on viral infection outcomes (Table 4). These changes involve viral proteins, host metabolic reprogramming, and stress signaling pathways, demonstrating diverse mechanisms of mitochondrial regulation during viral infection. At the molecular level, several studies described in Sections 2 and 3 have characterized specific protein domains, regions, residues, or sites involved in mitochondrial interactions or functional regulation [124, 126, 137, 145]. Specific interaction and functional regions/sites characterized at the domain or residue level are summarized in Table 5. Not all listed regions/sites have been confirmed as direct physical binding interfaces. These findings establish the functional significance of mitochondrial regulation during viral infection and provide a mechanistic rationale for further investigating mitochondrial pathways as potential antiviral targets.

**TABLE 5:** Molecular interactions and functional regions/sites involved in virus–Mitochondria regulation.

Virus	Viral protein	Mitochondrial target	Interaction/functional region or site	Experimental evidence	References
RSV	M	Mitochondrial gene transcription	M R170T/K172T	Mutagenesis; qPCR; Seahorse; MitoQ rescue	[62]
RABV	P	Complex I	P aa139–172	Deletion mapping; co‐IP; ROS assays	[124]
Mokola virus	M	Complex IV (COX1)	M aa46–110 (α2: aa69–82; K77/N81); COX1 aa104–125	Y2H; co‐IP; mutagenesis; CcO assay	[126]
HIV	p2 Peptide, Vpr	Complex IV (COX1), ANT/VDAC	p2 Gln6 ↔ COX1 N214–F235, Vpr aa52–96; aa71–82; R73/R77/R80	Y2H; Ala‐scanning; ATP assay, SPR	[137, 168]
HBV	HBx	Complex IV (COX3)	HBx aa72–117	Co‐IP; deletion mapping	[146]
HPV	E7	ATP5B	E7 L23	Y2H; co‐IP; mutagenesis; Seahorse	[158]
DENV	NS4B	DRP1 (Ser616 phosphorylation)	NS4B 2K sequence	p‐DRP1/CDK1 WB; deletion analysis	[170]
HCV	Core	Hsp60	Core aa1–75	Co‐IP; MS mapping; ROS assay	[175]
Influenza virus	PB1‐F2	ΔΨm	PB1‐F2 aa63–75 and aa69–82 (overlapping regions); K73/R75	Deletion mapping; mutagenesis; ΔΨm/apoptosis assays	[182, 183]

*Note*: Interaction/functional regions or sites indicate experimentally characterized protein regions or residues involved in direct molecular interactions or mitochondrial functional regulation and do not necessarily represent physical binding interfaces.

Abbreviations: Virus: RSV = respiratory syncytial virus, RABV = Rabies virus, HIV = human immunodeficiency virus, HBV = hepatitis B virus, HPV = human papillomavirus, DENV = dengue virus, HCV = hepatitis C virus. Viral protein: M = matrix protein, P = phosphoprotein, p2 peptide = Gag spacer peptide 1, Vpr = viral protein R, HBx = HBV X protein, E7 = early protein 7, NS4B = nonstructural protein 4B, Core = Capsid protein, PB1‐F2 = polymerase basic protein 1 ‐ Frame 2. Mitochondrial target: ANT = adenine nucleotide translocator, VDAC = voltage‐dependent anion channel, DRP1 = dynamin‐related protein‐1, Hsp60 = heat shock protein 60, ΔΨm = mitochondrial membrane potential. Experimental evidence: qPCR = quantitative polymerase chain reaction, MitoQ = mitoquinone mesylate, co‐IP = co‐immunoprecipitation, ROS = reactive oxygen species, Y2H = yeast two‐hybrid, CcO = cytochrome c oxidase, ATP = adenosine triphosphate, SPR = surface plasmon resonance, CDK1 = cyclin‐dependent kinase 1, MS = mass spectrometry.

## 4 Therapeutic Strategies Targeting Mitochondria in Viral Infections

As described in the previous sections, viral manipulation of mitochondrial metabolism, dynamics, mitophagy, and apoptosis is not merely an indirect effect of infection but often plays an active role in the viral life cycle. These mitochondrial processes therefore represent potential targets in therapeutic strategies targeting mitochondria‐associated host processes that influence viral infection outcomes. Current approaches include modulation of virus‐induced metabolic alterations, restoration of mitochondrial dynamics and quality control, modulation of mtROS production, and regulation of mitochondria‐dependent apoptotic signaling. Although most evidence currently derives from mechanistic studies and preclinical infection models, several mitochondria‐associated compounds and metabolic modulators have progressed into translational or clinical evaluation, providing a basis for evaluating mitochondria‐directed antiviral intervention.

### 4.1 Inhibiting Viral Strategies That Target Mitochondria

One potential therapeutic approach is to target virus‐specific mechanisms that directly modulate mitochondrial functions. Several viral proteins localize to mitochondria and remodel mitochondrial bioenergetics, dynamics, or antiviral signaling pathways to facilitate replication and persistence. For example, HBV HBx protein accumulates at mitochondria, alters ΔΨm, and interacts with mitochondrial proteins involved in respiratory regulation and mitophagy [190, 191]. HCV core protein interacts with Hsp60 and promotes ROS generation and mitochondrial dysfunction. The N‐terminal aa1‐75 region of the HCV core protein mediates its interaction with Hsp60, and deletion of this region abolishes Hsp60 binding and core‐induced ROS production, indicating that this interaction is functionally linked to oxidative stress [175]. Similarly, HPV E2 protein has been reported to interact with components of mitochondrial complex III, providing evidence of viral protein interactions with mitochondrial respiratory regulation [157]. These findings suggest that interactions between viral and mitochondrial components provide potential targets for antiviral strategies. Compared with broad inhibition of OXPHOS, disruption of specific protein‐protein interactions between viral proteins and mitochondrial components offers the potential to suppress viral replication while limiting systemic metabolic toxicity. This approach can involve inhibiting viral protein localization to mitochondria, disrupting specific interactions between viral and mitochondrial proteins, or targeting virus‐induced mitochondrial signaling complexes.

### 4.2 Modulating Mitochondrial Dynamics

Mitochondrial dynamics are increasingly recognized as therapeutically relevant during viral infection because several viruses alter mitochondrial morphology, positioning, and fusion–fission balance to support replication or evade antiviral responses. RSV infection promotes microtubule‐dependent mitochondrial repositioning and redistribution within infected cells, suggesting that mitochondrial trafficking contributes to viral replication and cellular remodeling [162]. HIV Vpr has been associated with degradation of Mfn2 and disruption of mitochondrial integrity, linking viral protein expression to altered mitochondrial dynamics and signaling related to apoptosis [166]. These findings suggest that viruses actively manipulate mitochondrial dynamics to optimize replication and immune evasion. Targeting mitochondrial dynamics can therefore represent a selective host‐directed antiviral strategy. Pharmacological modulation of excessive mitochondrial fission, including approaches using DRP1‐targeting compounds such as Mdivi‐1, has been reported to preserve mitochondrial integrity and attenuate virus‐associated mitochondrial dysfunction in experimental systems. Conversely, restoration of mitochondrial fusion through modulation of Mfn1/2 activity can improve antiviral signaling and limit virus‐associated mitochondrial remodeling. Compared with broad inhibition of OXPHOS, therapeutic modulation of mitochondrial dynamics can provide a more selective approach to interfere with infection‐associated mitochondrial alterations while preserving essential bioenergetic function.

### 4.3 Mitochondrial Antioxidants and Metabolic Modulators

Because mtROS production and metabolic alterations are frequently observed in viral infection, mitochondrial antioxidants and metabolic modulators have emerged as potential host‐directed antiviral strategies. Viral infection frequently disrupts mitochondrial redox balance and central metabolic pathways to support replication and persistence, suggesting that restoration of metabolic homeostasis offers a potential strategy for interfering with virus‐associated mitochondrial remodeling. RSV infection increases mtROS accumulation, and treatment with the mitochondria‐targeted antioxidant MitoQ has been shown to reduce mtROS production, suppress RSV genome replication, and attenuate airway inflammation in both epithelial cell systems and mouse models [61]. SARS‐CoV‐2 infection is also associated with oxidative stress and metabolic alterations characterized by glycolytic and inflammatory phenotypes, while influenza viruses show subtype‐dependent metabolic regulation during infection [192]. Metabolic modulators targeting mitochondrial pathways have also demonstrated antiviral potential in experimental systems. Metformin, which suppresses mitochondrial complex I activity and regulates AMPK‐associated metabolic signaling, has been associated with reduced HIV replication and altered viral persistence‐related metabolism in infected cells [135]. Dimethyl malonate, an inhibitor of SDH, has demonstrated antiviral and immunometabolic effects in HBV‐ and SARS‐CoV‐2‐related studies. These effects are associated with modulation of mitochondrial metabolism and inflammatory signaling. This supports selective targeting of mitochondrial metabolic pathways as an approach to alter infection‐associated host responses [103, 148]. Mitochondrial antioxidants and metabolic modulators can provide therapeutic benefit through regulation of mtROS, immunometabolism, and infection‐associated metabolic alterations. Because mitochondrial metabolism is essential for normal cellular function, therapeutic strategies targeting these pathways will require careful evaluation of toxicity, tissue specificity, and infection stage‐dependent metabolic effects.

### 4.4 Regulating Mitophagy

Mitophagy is increasingly recognized as a therapeutically relevant process during viral infection. Several viruses manipulate mitochondrial quality control pathways to support replication, persistence, or immune evasion. Virus‐induced mitophagy removes damaged mitochondria, suppresses MAVS, and facilitates adaptation to metabolic stress during infection. HCV NS5A promotes Parkin‐mediated mitophagy and alters mitochondrial dynamics and metabolic homeostasis during infection [177, 179]. These findings suggest that viruses can manipulate mitochondrial homeostasis to support intracellular conditions that support viral replication. Therapeutic modulation of mitophagy therefore requires a selective approach. Inhibition of virus‐induced mitophagy offers a potential strategy to suppress viral persistence and restore antiviral signaling. Conversely, controlled enhancement of mitophagy could facilitate the removal of damaged mitochondria, reduce mitochondrial stress, and limit inflammatory tissue injury during severe infection. Accordingly, selective regulation of mitophagy could limit mitochondrial dysfunction during infection while preserving essential mitochondrial quality control functions.

### 4.5 Targeting the Mitochondrial Apoptosis Pathway

Mitochondria‐associated apoptotic signaling also represents a potential therapeutic target during viral infection. Diverse viruses regulate mitochondrial membrane integrity and apoptotic signaling to influence cell survival, replication efficiency, and tissue injury during infection. Viral proteins can directly alter ΔΨm, induce cytochrome c release, or regulate mitochondrial permeability transition pathways to control cell survival during infection. Several viral proteins directly regulate mitochondrial apoptosis. HIV Tat and Vpr induce mitochondrial dysfunction and disrupt ΔΨm, followed by activation of apoptotic signaling pathways, with mitochondrial injury contributing to viral cytopathology [167, 168]. HBV HBx is also involved in mitochondrial remodeling and cytochrome c release, supporting involvement of mitochondrial apoptosis signaling during infection [24, 185]. In addition, influenza PB1‐F2 and Mokola virus M protein affect mitochondrial membrane integrity and respiratory components, further supporting their involvement in cell death during infection [126, 163]. Therapeutic targeting of mitochondrial apoptosis pathways therefore offers selective opportunities to limit viral pathogenesis and tissue injury. Because viruses can either suppress or induce apoptosis depending on infection stage and viral replication requirements, therapeutic modulation of mitochondrial apoptosis pathways requires virus‐ and stage‐specific approaches.

### 4.6 Mitochondria‐Targeted Compounds in Translational and Clinical Studies

Among mitochondria‐directed agents evaluated in viral infection studies, MitoQ is one of the most extensively investigated mitochondrial antioxidants. MitoQ consists of a triphenylphosphonium (TPP^+^)‐conjugated ubiquinone structure that preferentially accumulates within mitochondria because of the highly negative ΔΨm [193]. Preclinical studies demonstrated that MitoQ reduces mitochondrial oxidative stress and improves infection‐associated inflammatory outcomes in RSV models [61, 162]. MitoQ has demonstrated acceptable safety and tolerability profiles in a human study outside antiviral settings [194]. MitoQ has also been evaluated in an exploratory clinical study involving individuals exposed to SARS‐CoV‐2 (ClinicalTrials.gov identifier: NCT05381454). However, antiviral efficacy specifically resulting from mitochondrial targeting in humans has not been clearly established. Metformin, a host‐directed metabolic regulator associated with mitochondrial complex I inhibition and AMPK mechanistic target of rapamycin (mTOR) modulation, has also been investigated in relation to viral diseases [195]. Because viral replication frequently depends on metabolic alterations and mitochondrial adaptation to sustain replication, modulation of host immunometabolism has emerged as a potential antiviral strategy. The Phase III COVID‐OUT trial evaluating metformin in SARS‐CoV‐2 infection (ClinicalTrials.gov identifier: NCT04510194) reported a reduction in the composite outcome of emergency department visit, hospitalization, or death, although the precise contribution of mitochondrial mechanisms has not been fully determined. These clinical benefits should be distinguished from direct antiviral effects. These findings support further investigation of host metabolic modulation as a therapeutic approach during viral infection. Accumulating evidence identifies mitochondrial processes as important host dependencies during viral infection. Therapeutic development therefore requires selective modulation of virus‐induced mitochondrial remodeling while preserving essential host bioenergetic and immunological functions. Combination approaches integrating mitochondria‐directed agents with direct‐acting antivirals or immunomodulatory therapies offer the potential to improve antiviral efficacy while limiting systemic toxicity.

## 5 Challenges and Future Directions

Despite increasing evidence that viruses regulate mitochondrial OXPHOS and associated mitochondrial functions, several challenges continue to limit mechanistic interpretation and clinical translation. Current studies show considerable variation in mitochondrial responses depending on virus type, infection stage, host cell, and experimental model [17, 18, 36, 196]. Distinguishing mitochondrial processes that directly affect viral infection outcomes from secondary metabolic alterations remains difficult [7, 8, 31, 36]. Addressing these limitations requires experimental strategies that establish direct functional effects, incorporate physiologically relevant models, identify virus‐specific mitochondrial dependencies, and facilitate the translation of mechanistic findings into antiviral strategies.

### 5.1 Distinguishing Direct Viral Regulation From Infection‐Induced Host Responses

A major unresolved question is whether mitochondrial alterations during infection result from direct viral regulation or from host responses induced by viral infection. Viral proteins can directly interact with mitochondrial proteins and OXPHOS components, supporting direct regulation of host mitochondrial functions by viruses [7, 8, 9, 197]. However, mitochondrial dysfunction can also arise indirectly from immune activation, oxidative stress, calcium imbalance, and metabolic changes induced by infection [9, 17, 18, 19]. These mechanisms are not mutually exclusive, and their relative contributions can vary depending on virus type, infection stage, and host conditions [17, 18, 31, 36]. Infection‐associated changes in mitochondrial respiration, membrane potential, ROS production, or mitochondrial morphology do not necessarily demonstrate a functional contribution to viral infection. Determining these functional effects therefore requires experimental manipulation of the affected mitochondrial pathway followed by measurement of viral outcomes, including viral genome replication, viral protein expression, and infectious virion production. Analysis at different stages of infection is also important for distinguishing early virus‐induced mitochondrial events from later host‐mediated responses. Time‐course metabolic profiling combined with genetic manipulation of ETC components or mitochondrial regulators can help determine their functional effects on viral infection outcomes.

### 5.2 Experimental Models and Methodological Limitations

Interpretation of mitochondrial phenotypes is also constrained by current experimental approaches. Many studies rely on pharmacological inhibitors of OXPHOS, including rotenone and oligomycin. Although these compounds are useful for identifying mitochondrial dependencies, respiratory chain inhibitors can induce effects beyond inhibition of their intended ETC targets, thereby complicating interpretation of functional outcomes [11, 12, 198, 199]. Genetic manipulation of specific ETC subunits or mitochondrial regulators, combined with rescue experiments, enables validation of pharmacologically identified mitochondrial dependencies. Another limitation is the extensive use of immortalized cell lines, which can exhibit mitochondrial and metabolic characteristics that differ from primary cells and tissues [31, 36, 200]. Mitochondrial responses observed in these experimental systems do not necessarily represent those occurring during infection in vivo. Future studies should incorporate primary cells, organoids, differentiated cell systems, and in vivo models to determine whether mitochondrial phenotypes identified in cell lines are conserved under physiologically relevant conditions. Live‐cell analysis of mitochondrial respiration, membrane potential, and mitochondrial dynamics can provide temporal information that cannot be obtained from endpoint measurements. Transcriptomic, proteomic, and metabolomic approaches have substantially expanded the characterization of virus‐induced metabolic alterations [18, 30, 35, 201]. However, integration of these datasets with direct functional measurements of mitochondrial activity remains limited. Combining multi‐omics approaches with respirometry, isotope tracing analyses, mitochondrial imaging, and genetic manipulation enables the distinction between correlative metabolic signatures and mitochondrial processes that directly affect infection. Spatial metabolomics is emerging as a useful approach for investigating tissue‐level metabolic heterogeneity during viral infection [202, 203, 204]. Integrating this approach with viral localization and functional mitochondrial assays may help clarify the relationship between local metabolic alterations and mitochondrial responses during viral infection.

### 5.3 Identifying Virus‐Specific Mitochondrial Dependencies for Therapeutic Targeting

Mitochondrial bioenergetics represents a potential antiviral target, but the diversity of mitochondrial responses among viruses limits the applicability of broad OXPHOS inhibition [13, 17, 36, 196]. Enhanced OXPHOS activity supports viral infection in several experimental systems, whereas other viruses suppress individual ETC complexes or induce mitochondrial dysfunction. The effects of targeting mitochondrial pathways depend on the virus and experimental conditions and cannot be generalized across viral infections [17, 36, 196]. Future studies should therefore identify virus‐specific mitochondrial dependencies at the level of individual ETC complexes, subunits, metabolic pathways, and mitochondrial regulatory proteins. Testable questions include whether individual ETC complexes are required at specific stages of viral replication, whether viral proteins interact directly with individual OXPHOS subunits, and whether disruption of these interactions can suppress viral replication without disrupting essential cellular bioenergetic functions. Virus‐specific mitochondrial dependencies are not limited to OXPHOS. ROS signaling, apoptosis, calcium flux, mitochondrial dynamics, and mitochondria‐associated antiviral signaling represent additional intervention points [2, 9, 31, 205]. The distinction between functionally validated mitochondrial dependencies and mitochondrial alterations observed during infection can also guide the selection of therapeutic targets. Mitochondrial processes for which pharmacological or genetic manipulation produces reproducible changes in viral genome replication, viral protein expression, or infectious virion production warrant further mechanistic validation and preclinical evaluation.

### 5.4 Bridging Mechanistic Studies and Clinical Translation

Translation of mitochondria targeting strategies into antiviral therapies remains challenging because mitochondria are essential for normal cellular physiology. Although modulation of mitochondrial metabolism can suppress viral replication in experimental systems, broad targeting of host bioenergetics raises potential concerns regarding toxicity and tissue‐specific metabolic vulnerability [13, 36, 193, 195]. Therapeutic development should therefore focus on virus‐specific mitochondrial interactions or regulatory pathways rather than nonselective suppression of mitochondrial respiration. This limitation is evident in studies of MitoQ, a mitochondria‐targeted antioxidant that underwent a phase II clinical study in patients with chronic HCV infection and reduced serum aminotransferase levels but did not significantly alter HCV viral load. This discrepancy between improved biochemical markers and unchanged viral load reflects the difficulty of translating mitochondrial or oxidative stress‐associated effects into direct antiviral efficacy [206]. Successful clinical translation requires connecting molecular mitochondrial mechanisms with viral outcomes and disease phenotypes. Mitochondrial dysfunction during viral infection can influence innate immunity, inflammatory responses, apoptosis, and cellular metabolic homeostasis [2, 3, 31, 197]. Determining whether mitochondrial modulation directly affects viral infection or results from host responses induced by infection is therefore essential for developing mitochondria‐directed therapeutic strategies. Future studies should determine therapeutic windows, tissue‐specific effects, and whether mitochondrial dependencies identified in experimental systems are conserved in human infection. Integrating single‐cell metabolic profiling with functional assays in primary cells, organoids, and in vivo systems provides a means to identify cell type‐specific mitochondrial dependencies and facilitate the translation of mechanistic findings into clinically relevant strategies.

## 6 Conclusion

Mitochondrial functions are altered during viral infection and influence viral replication, antiviral signaling, cellular stress responses, and virus‐associated cellular outcomes. Viral proteins can directly target OXPHOS components and mitochondrial proteins, while infection can also induce alterations in mitochondrial metabolism, bioenergetics, ROS signaling, apoptosis, calcium homeostasis, mitochondrial dynamics, and innate immune signaling. The functional effects of these mitochondrial alterations differ across viruses and experimental systems. Distinguishing mitochondrial processes that directly affect viral infection outcomes from mitochondrial changes observed during infection is therefore essential for interpreting current evidence and identifying therapeutically relevant targets. Future studies integrating genetic manipulation, quantitative mitochondrial assays, time‐course analyses, physiologically relevant infection models, and multi‐omics approaches are needed to identify virus‐specific mitochondrial dependencies and validate their functional effects on viral infection. A detailed understanding of these dependencies provides a foundation for developing antiviral strategies that selectively target mitochondrial processes involved in viral infection while preserving essential host mitochondrial functions.

## Author Contributions

H.Y.O. and H.J.S. conceptualized and wrote the manuscript. H.Y.O. drew the figures and summarized the data in the tables. H.J.S. guided and supervised the study. All the authors contributed to the article and approved the manuscript for publication.

## Funding

This work was supported by the National Research Foundation of Korea (NRF) grant funded by the Korean Government (MSIT) (No. RS‐2025‐02263643, RS‐2026‐25501101), and the BK 21 FOUR Program by the Chungnam National University Research Grant, 2025.

## Ethics Statement

The authors have nothing to report.

## Conflicts of Interest

The authors declare no conflicts of interest.

## Data Availability

The authors have nothing to report.

## References

[1] B. Banoth and S. L. Cassel , “Mitochondria in Innate Immune Signaling,” Translational Research 202 (2018): 52–68. 10.1016/j.trsl.2018.07.014 PMC6218307 30165038

[2] K. Yasukawa and T. Koshiba , “Mitochondrial Reactive Zones in Antiviral Innate Immunity,” Biochimica et Biophysica Acta—General Subjects 1865 (2021): 129839. 10.1016/j.bbagen.2020.129839 33412226

[3] A. Mohanty , R. Tiwari‐Pandey , and N. R. Pandey , “Mitochondria: The Indispensable Players in Innate Immunity and Guardians of the Inflammatory Response,” Journal of Cell Communication and Signaling 13 (2019): 303–318. 10.1007/s12079-019-00507-9 PMC6732146 30719617

[4] H. S. Jin , H. W. Suh , S. J. Kim , et al., “Mitochondrial Control of Innate Immunity and Inflammation,” Immune Network 17 (2017): 77–88. 10.4110/in.2017.17.2.77 PMC5407986 28458619

[5] D. Arnoult , L. Carneiro , I. Tattoli , et al., “The Role of Mitochondria in Cellular Defense Against Microbial Infection,” Seminars in Immunology 21 (2009): 223–232. 10.1016/j.smim.2009.05.009 19535268

[6] C. A. Benedict , P. S. Norris , and C. F. Ware , “To Kill or Be Killed: Viral Evasion of Apoptosis,” Nature Immunology 3 (2002): 1013–1018. 10.1038/ni1102-1013 12407409

[7] C. Claus and U. G. Liebert , “A Renewed Focus on the Interplay Between Viruses and Mitochondrial Metabolism,” Archives of Virology 159 (2014): 1267–1277. 10.1007/s00705-013-1841-1 24343264

[8] R. Saxena , P. Sharma , S. Kumar , et al., “Modulation of Mitochondria by Viral Proteins,” Life Sciences 313 (2023): 121271. 10.1016/j.lfs.2022.121271 36526048

[9] M. Khan , G. H. Syed , S. J. Kim , et al., “Mitochondrial Dynamics and Viral Infections: A Close Nexus,” Biochimica et Biophysica Acta (BBA)—Molecular Cell Research 1853 (2015): 2822–2833. 10.1016/j.bbamcr.2014.12.040 PMC4500740 25595529

[10] M. Pourcelot and D. Arnoult , “Mitochondrial Dynamics and the Innate Antiviral Immune Response,” FEBS Journal 281 (2014): 3791–3802. 10.1111/febs.12940 25051991

[11] R. Z. Zhao , S. Jiang , L. Zhang , et al., “Mitochondrial Electron Transport Chain, ROS Generation and Uncoupling (Review),” International Journal of Molecular Medicine 44 (2019): 3–15. 10.3892/ijmm.2019.4188 PMC6559295 31115493

[12] D. Nolfi‐Donegan , A. Braganza , and S. Shiva , “Mitochondrial Electron Transport Chain: Oxidative Phosphorylation, Oxidant Production, and Methods of Measurement,” Redox Biology 37 (2020): 101674. 10.1016/j.redox.2020.101674 PMC7767752 32811789

[13] T. El‐Bacha and A. T. Da Poian , “Virus‐Induced Changes in Mitochondrial Bioenergetics as Potential Targets for Therapy,” International Journal of Biochemistry and Cell Biology 45 (2013): 41–46. 10.1016/j.biocel.2012.09.021 23036789

[14] M. Rigoulet , E. D. Yoboue , and A. Devin , “Mitochondrial Ros Generation and Its Regulation: Mechanisms Involved in H(2)O(2) Signaling,” Antioxidants & Redox Signaling 14 (2011): 459–468. 10.1089/ars.2010.3363 20649461

[15] R. J. Mailloux , S. L. McBride , and M. E. Harper , “Unearthing the Secrets of Mitochondrial ROS and Glutathione in Bioenergetics,” Trends in Biochemical Sciences 38 (2013): 592–602. 10.1016/j.tibs.2013.09.001 24120033

[16] E. K. Hui and D. P. Nayak , “Role of ATP in Influenza Virus Budding,” Virology 290 (2001): 329–341. 10.1006/viro.2001.1181 11883197

[17] E. L. Sanchez and M. Lagunoff , “Viral Activation of Cellular Metabolism,” Virology 479–480 (2015): 609–618. 10.1016/j.virol.2015.02.038 PMC4424078 25812764

[18] K. Girdhar , A. Powis , A. Raisingani , et al., “Viruses and Metabolism: The Effects of Viral Infections and Viral Insulins on Host Metabolism,” Annual Review of Virology 8 (2021): 373–391. 10.1146/annurev-virology-091919-102416 PMC9175272 34586876

[19] C. N. S. Allen , S. P. Arjona , M. Santerre , et al., “Hallmarks of Metabolic Reprogramming and Their Role in Viral Pathogenesis,” Viruses 14 (2022): 602. 10.3390/v14030602 PMC8955778 35337009

[20] L. Yi , J. Song , Z. Zhang , et al., “Palmitoyl‐Transferase 3 Promotes Mitochondrial Antiviral Signaling Protein Degradation by Modulating Its Ubiquitination,” International Journal of Biological Macromolecules 310 (2025): 143609. 10.1016/j.ijbiomac.2025.143609 40300684

[21] H. Zhu and C. Zheng , “The Race Between Host Antiviral Innate Immunity and the Immune Evasion Strategies of Herpes Simplex Virus 1,” Microbiology and Molecular Biology Reviews 84 (2020): e00099–20. 10.1128/MMBR.00099-20 PMC7528619 32998978

[22] C. Su , G. Zhan , and C. Zheng , “Evasion of Host Antiviral Innate Immunity by HSV‐1, an Update,” Virology Journal 13 (2016): 38. 10.1186/s12985-016-0495-5 PMC4782282 26952111

[23] P. Boya , T. Roumier , K. Andreau , et al., “Mitochondrion‐Targeted Apoptosis Regulators of Viral Origin,” Biochemical and Biophysical Research Communications 304 (2003): 575–581. 10.1016/s0006-291x(03)00630-2 12729592

[24] D. Li , X. Z. Wang , J. P. Yu , et al., “Cytochrome C Oxidase III Interacts With Hepatitis B Virus X Protein in Vivo by Yeast Two‐Hybrid System,” World Journal of Gastroenterology 10 (2004): 2805–2808. 10.3748/wjg.v10.i19.2805 PMC4572106 15334674

[25] A. Soucy‐Faulkner , E. Mukawera , K. Fink , et al., “Requirement of Nox2 and Reactive Oxygen Species for Efficient Rig‐I‐Mediated Antiviral Response Through Regulation of MAVS Expression,” PLOS Pathogens 6 (2010): e1000930. 10.1371/journal.ppat.1000930 PMC2880583 20532218

[26] L. Galluzzi , C. Brenner , E. Morselli , et al., “Viral Control of Mitochondrial Apoptosis,” PLOS Pathogens 4 (2008): e1000018. 10.1371/journal.ppat.1000018 PMC2376094 18516228

[27] K. Yasukawa , H. Oshiumi , M. Takeda , et al., “Mitofusin 2 Inhibits Mitochondrial Antiviral Signaling,” Science Signaling 2 (2009): ra47. 10.1126/scisignal.2000287 19690333

[28] K. Onoguchi , K. Onomoto , S. Takamatsu , et al., “Virus‐Infection or 5'ppp‐RNA Activates Antiviral Signal Through Redistribution of IPS‐1 Mediated by Mfn1,” PLOS Pathogens 6 (2010): e1001012. 10.1371/journal.ppat.1001012 PMC2908619 20661427

[29] Q. Zhou , D. Shi , Y. D. Tang , et al., “Pseudorabies Virus Gm and Its Homologous Proteins in Herpesviruses Induce Mitochondria‐Related Apoptosis Involved in Viral Pathogenicity,” PLOS Pathogens 20 (2024): e1012146. 10.1371/journal.ppat.1012146 PMC11051632 38669242

[30] B. Shen , X. Yi , Y. Sun , et al., “Proteomic and Metabolomic Characterization of COVID‐19 Patient Sera,” Cell 182 (2020): 59–72.e15. 10.1016/j.cell.2020.05.032 PMC7254001 32492406

[31] M. Sorouri , T. Chang , and D. C. Hancks , “Mitochondria and Viral Infection: Advances and Emerging Battlefronts,” MBio 13 (2022): e0209621. 10.1128/mbio.02096-21 PMC8787485 35073751

[32] A. Lercher , A. Bhattacharya , A. M. Popa , et al., “Type I Interferon Signaling Disrupts the Hepatic Urea Cycle and Alters Systemic Metabolism to Suppress T Cell Function,” Immunity 51 (2019): 1074–1087.e9. 10.1016/j.immuni.2019.10.014 PMC6926485 31784108

[33] X. K. Li , Q. B. Lu , W. W. Chen , et al., “Arginine Deficiency Is Involved in Thrombocytopenia and Immunosuppression in Severe Fever With Thrombocytopenia Syndrome,” Science Translational Medicine 10 (2018): eaat4162. 10.1126/scitranslmed.aat4162 30232226

[34] S. K. Thaker , T. Chapa , G. Garcia Jr. , et al., “Differential Metabolic Reprogramming by Zika Virus Promotes Cell Death in Human versus Mosquito Cells,” Cell Metabolism 29 (2019): 1206–1216.e4. 10.1016/j.cmet.2019.01.024 PMC6818653 30827860

[35] D. Bojkova , K. Klann , B. Koch , et al., “Proteomics of SARS‐CoV‐2‐Infected Host Cells Reveals Therapy Targets,” Nature 583 (2020): 469–472. 10.1038/s41586-020-2332-7 PMC7616921 32408336

[36] N. Purandare , E. Ghosalkar , L. I. Grossman , et al., “Mitochondrial Oxidative Phosphorylation in Viral Infections,” Viruses 15 (2023): 2380. 10.3390/v15122380 PMC10747082 38140621

[37] S. Elesela and N. W. Lukacs , “Role of Mitochondria in Viral Infections,” Life 11 (2021): 232. 10.3390/life11030232 PMC7998235 33799853

[38] P. K. Das and M. Kielian , “Molecular and Structural Insights Into the Life Cycle of Rubella Virus,” Journal of Virology 95 (2021): e02349–20. 10.1128/JVI.02349-20 PMC8139664 33627388

[39] M. Sakata and Y. Mori , “[the Life Cycle of Rubella Virus],” Uirusu 64 (2014): 137–146. 10.2222/jsv.64.137 26437836

[40] S. George , R. Viswanathan , and G. N. Sapkal , “Molecular Aspects of the Teratogenesis of Rubella Virus,” Biological Research 52 (2019): 47. 10.1186/s40659-019-0254-3 PMC6712747 31455418

[41] C. D. Atreya , K. V. Mohan , and S. Kulkarni , “Rubella Virus and Birth Defects: Molecular Insights Into the Viral Teratogenesis at the Cellular Level,” Birth Defects Research Part A: Clinical and Molecular Teratology 70 (2004): 431–437. 10.1002/bdra.20045 15259032

[42] J. Fontana , C. López‐Iglesias , W. P. Tzeng , et al., “Three‐Dimensional Structure of Rubella Virus Factories,” Virology 405 (2010): 579–591. 10.1016/j.virol.2010.06.043 PMC7111912 20655079

[43] C. Claus , S. Chey , S. Heinrich , et al., “Involvement of P32 and Microtubules in Alteration of Mitochondrial Functions by Rubella Virus,” Journal of Virology 85 (2011): 3881–3892. 10.1128/JVI.02492-10 PMC3126120 21248045

[44] C. Claus , K. Schonefeld , D. Hubner , et al., “Activity Increase in Respiratory Chain Complexes by Rubella Virus With Marginal Induction of Oxidative Stress,” Journal of Virology 87 (2013): 8481–8492. 10.1128/JVI.00533-13 PMC3719815 23720730

[45] F. S. Garmaroudi , D. Marchant , R. Hendry , et al., “Coxsackievirus B3 Replication and Pathogenesis,” Future Microbiology 10 (2015): 629–653. 10.2217/fmb.15.5 25865198

[46] X. Li , M. Wang , A. Cheng , et al., “Enterovirus Replication Organelles and Inhibitors of Their Formation,” Frontiers in Microbiology 11 (2020): 1817. 10.3389/fmicb.2020.01817 PMC7468505 32973693

[47] K. U. Knowlton , “Cvb Infection and Mechanisms of Viral Cardiomyopathy,” Current Topics in Microbiology and Immunology 323 (2008): 315–335. 10.1007/978-3-540-75546-3_15 18357777

[48] P. E. Tam , “Coxsackievirus Myocarditis: Interplay Between Virus and Host in the Pathogenesis of Heart Disease,” Viral Immunology 19 (2006): 133–146. 10.1089/vim.2006.19.133 16817756

[49] C. Leipner , K. Grün , I. Schneider , et al., “Coxsackievirus B3‐Induced Myocarditis: Differences in the Immune Response of C57bl/6 and Balb/C Mice,” Medical Microbiology and Immunology 193 (2004): 141–147. 10.1007/s00430-003-0199-5 14593475

[50] L. J. Wolfgram , K. W. Beisel , A. Herskowitz , et al., “Variations in the Susceptibility to Coxsackievirus B3‐Induced Myocarditis Among Different Strains of Mice,” Journal of Immunology 136 (1986): 1846–1852. 3005402

[51] L. Ebermann , S. Wika , I. Klumpe , et al., “The Mitochondrial Respiratory Chain Has a Critical Role in the Antiviral Process in Coxsackievirus B3‐Induced Myocarditis,” Laboratory Investigation 92 (2012): 125–134. 10.1038/labinvest.2011.145 21968812

[52] R. LeDesma , I. Nimgaonkar , and A. Ploss , “Hepatitis E Virus Replication,” Viruses 11 (2019): 719. 10.3390/v11080719 PMC6723718 31390784

[53] X. Zhou , F. Huang , L. Xu , et al., “Hepatitis E Virus Infects Neurons and Brains,” Journal of Infectious Diseases 215 (2017): 1197–1206. 10.1093/infdis/jix079 28199701

[54] C. Qu , S. Zhang , W. Wang , et al., “Mitochondrial Electron Transport Chain Complex III Sustains Hepatitis E Virus Replication and Represents an Antiviral Target,” FASEB Journal 33 (2019): 1008–1019. 10.1096/fj.201800620R PMC6355081 30070932

[55] A. Pierangeli , C. Scagnolari , and G. Antonelli , “Respiratory Syncytial Virus,” Minerva Pediatrica 70 (2018): 553–565. 10.23736/S0026-4946.18.05312-4 30334622

[56] L. Glaser , P. J. Coulter , M. Shields , et al., “Airway Epithelial Derived Cytokines and Chemokines and Their Role in the Immune Response to Respiratory Syncytial Virus Infection,” Pathogens 8 (2019): 106. 10.3390/pathogens8030106 PMC6789711 31331089

[57] P. Hillyer , R. Shepard , M. Uehling , et al., “Differential Responses by Human Respiratory Epithelial Cell Lines to Respiratory Syncytial Virus Reflect Distinct Patterns of Infection Control,” Journal of Virology 92 (2018): e02202–2217. 10.1128/JVI.02202-17 PMC6052282 29769339

[58] L. Broadbent , R. Villenave , H. Guo‐Parke , et al., “In Vitro Modeling of RSV Infection and Cytopathogenesis in Well‐Differentiated Human Primary Airway Epithelial Cells (WD‐PAECs),” Methods in Molecular Biology 1442 (2016): 119–139. 10.1007/978-1-4939-3687-8_9 27464691

[59] M. Hu , H. M. Li , M. A. Bogoyevitch , et al., “Mitochondrial Protein P32/Hapb1/Gc1qr/C1qbp Is Required for Efficient Respiratory Syncytial Virus Production,” Biochemical and Biophysical Research Communications 489 (2017): 460–465. 10.1016/j.bbrc.2017.05.171 28576489

[60] S. Kim , H. Y. Kim , S. Lee , et al., “Hepatitis B Virus X Protein Induces Perinuclear Mitochondrial Clustering in Microtubule‐ and Dynein‐Dependent Manners,” Journal of Virology 81 (2007): 1714–1726. 10.1128/JVI.01863-06 PMC1797565 17151129

[61] M. Hu , M. A. Bogoyevitch , and D. A. Jans , “Subversion of Host Cell Mitochondria by RSV to Favor Virus Production Is Dependent on Inhibition of Mitochondrial Complex I and ROS Generation,” Cells 8 (2019): 1417. 10.3390/cells8111417 PMC6912631 31717900

[62] M. Hu , M. A. Bogoyevitch , and D. A. Jans , “Respiratory Syncytial Virus Matrix Protein Is Sufficient and Necessary to Remodel Host Mitochondria in Infection,” Cells 12 (2023): 1311. 10.3390/cells12091311 PMC10177070 37174711

[63] P. M. Jean Beltran and I. M. Cristea , “The Life Cycle and Pathogenesis of Human Cytomegalovirus Infection: Lessons From Proteomics,” Expert Review of Proteomics 11 (2014): 697–711. 10.1586/14789450.2014.971116 PMC4604749 25327590

[64] H. L. Fulkerson , M. T. Nogalski , D. Collins‐McMillen , et al., “Overview of Human Cytomegalovirus Pathogenesis,” Methods in Molecular Biology 2244 (2021): 1–18. 10.1007/978-1-0716-1111-1_1 33555579

[65] P. Griffiths and M. Reeves , “Pathogenesis of Human Cytomegalovirus in the Immunocompromised Host,” Nature Reviews Microbiology 19 (2021): 759–773. 10.1038/s41579-021-00582-z PMC8223196 34168328

[66] Y. Xia , J. Zhang , X. Shui , et al., “Recent Advances in Human Cytomegalovirus: A Comprehensive Review of Pathogenic Mechanisms, Virus‐Host Interactions, and Antiviral Strategies,” Frontiers in Immunology 16 (2025): 1636978. 10.3389/fimmu.2025.1636978 PMC12855148 41624836

[67] Z. Shang and X. Li , “Human Cytomegalovirus: Pathogenesis, Prevention, and Treatment,” Molecular Biomedicine 5 (2024): 61. 10.1186/s43556-024-00226-7 PMC11589059 39585514

[68] L. Hertel and E. S. Mocarski , “Global Analysis of Host Cell Gene Expression Late During Cytomegalovirus Infection Reveals Extensive Dysregulation of Cell Cycle Gene Expression and Induction of Pseudomitosis Independent of Us28 Function,” Journal of Virology 78 (2004): 11988–12011. 10.1128/JVI.78.21.11988-12011.2004 PMC523267 15479839

[69] S. Karniely , M. P. Weekes , R. Antrobus , et al., “Human Cytomegalovirus Infection Upregulates the Mitochondrial Transcription and Translation Machineries,” MBio 7 (2016): e00029. 10.1128/mBio.00029-16 PMC4807356 27025248

[70] M. Kaarbo , E. Ager‐Wick , P. O. Osenbroch , et al., “Human Cytomegalovirus Infection Increases Mitochondrial Biogenesis,” Mitochondrion 11 (2011): 935–945. 10.1016/j.mito.2011.08.008 21907833

[71] H. M. Wilkins , S. M. Carl , and R. H. Swerdlow , “Cytoplasmic Hybrid (Cybrid) Cell Lines as a Practical Model for Mitochondriopathies,” Redox Biology 2 (2014): 619–631. 10.1016/j.redox.2014.03.006 PMC4297942 25460729

[72] J. A. Combs , E. B. Norton , Z. R. Saifudeen , et al., “Human Cytomegalovirus Alters Host Cell Mitochondrial Function During Acute Infection,” Journal of Virology 94 (2020): e01183–19. 10.1128/JVI.01183-19 PMC6955246 31694945

[73] C. N. Betsinger , C. S. R. Jankowski , W. A. Hofstadter , et al., “The Human Cytomegalovirus Protein Pul13 Targets Mitochondrial Cristae Architecture to Increase Cellular Respiration During Infection,” Proceedings of the National Academy of Sciences of the United States of America 118 (2021): e2101675118. 10.1073/pnas.2101675118 PMC8364163 34344827

[74] B. Moss , “Poxvirus Membrane Biogenesis,” Virology 479–480 (2015): 619–626. 10.1016/j.virol.2015.02.003 PMC4424062 25728299

[75] C. W. Chang , H. C. Li , C. F. Hsu , et al., “Increased ATP Generation in the Host Cell Is Required for Efficient Vaccinia Virus Production,” Journal of Biomedical Science 16 (2009): 80. 10.1186/1423-0127-16-80 PMC2741444 19725950

[76] K. A. Fontaine , R. Camarda , and M. Lagunoff , “Vaccinia Virus Requires Glutamine but Not Glucose for Efficient Replication,” Journal of Virology 88 (2014): 4366–4374. 10.1128/JVI.03134-13 PMC3993723 24501408

[77] A. Dai , S. Cao , P. Dhungel , et al., “Ribosome Profiling Reveals Translational Upregulation of Cellular Oxidative Phosphorylation mRNAs During Vaccinia Virus‐Induced Host Shutoff,” Journal of Virology 91 (2017): e01858–16. 10.1128/JVI.01858-16 PMC5309933 28003488

[78] C. Zhuo , D. Zheng , Z. He , et al., “Hsv‐1 Enhances the Energy Metabolism of Human Umbilical Cord Mesenchymal Stem Cells to Promote Virus Infection,” Future Virology 12 (2017): 349–360.

[79] A. Dasgupta and D. W. Wilson , “ATP Depletion Blocks Herpes Simplex Virus DNA Packaging and Capsid Maturation,” Journal of Virology 73 (1999): 2006–2015. 10.1128/jvi.73.3.2006-2015.1999 PMC104443 9971781

[80] S. Q. Zheng , Y. X. Li , Y. Zhang , et al., “miR‐101 Regulates HSV‐1 Replication by Targeting Atp5b,” Antiviral Research 89 (2011): 219–226. 10.1016/j.antiviral.2011.01.008 21291913

[81] K. Tabata , C. J. Neufeldt , and R. Bartenschlager , “Hepatitis C Virus Replication,” Cold Spring Harbor Perspectives in Medicine 10 (2020): a037093. 10.1101/cshperspect.a037093 PMC7050578 31570388

[82] P. S. Sung , V. Racanelli , and E. C. Shin , “CD8 (+) T‐Cell Responses in Acute Hepatitis C Virus Infection,” Frontiers in Immunology 5 (2014): 266. 10.3389/fimmu.2014.00266 PMC4047488 24936203

[83] M. Hofmann , C. Tauber , N. Hensel , et al., “CD8 (+) T Cell Responses During HCV Infection and HCC,” Journal of Clinical Medicine 10 (2021): 991. 10.3390/jcm10050991 PMC7957882 33801203

[84] M. Okuda , K. Li , M. R. Beard , et al., “Mitochondrial Injury, Oxidative Stress, and Antioxidant Gene Expression Are Induced by Hepatitis C Virus Core Protein,” Gastroenterology 122 (2002): 366–375. 10.1053/gast.2002.30983 11832451

[85] M. Korenaga , T. Wang , Y. Li , et al., “Hepatitis C Virus Core Protein Inhibits Mitochondrial Electron Transport and Increases Reactive Oxygen Species (ROS) Production,” Journal of Biological Chemistry 280 (2005): 37481–37488. 10.1074/jbc.M506412200 16150732

[86] C. Piccoli , R. Scrima , G. Quarato , et al., “Hepatitis C Virus Protein Expression Causes Calcium‐Mediated Mitochondrial Bioenergetic Dysfunction and Nitro‐Oxidative Stress,” Hepatology 46 (2007): 58–65. 10.1002/hep.21679 17567832

[87] G. Quarato , R. Scrima , M. Ripoli , et al., “Protective Role of Amantadine in Mitochondrial Dysfunction and Oxidative Stress Mediated by Hepatitis C Virus Protein Expression,” Biochemical Pharmacology 89 (2014): 545–556. 10.1016/j.bcp.2014.03.018 24726442

[88] H. R. Ramage , G. R. Kumar , E. Verschueren , et al., “A Combined Proteomics/Genomics Approach Links Hepatitis C Virus Infection With Nonsense‐Mediated mRNA Decay,” Molecular Cell 57 (2015): 329–340. 10.1016/j.molcel.2014.12.028 PMC4305532 25616068

[89] V. Barili , P. Fisicaro , B. Montanini , et al., “Targeting P53 and Histone Methyltransferases Restores Exhausted Cd8+ T Cells in HCV Infection,” Nature Communications 11 (2020): 604. 10.1038/s41467-019-14137-7 PMC6992697 32001678

[90] G. K. Gerresheim , J. Bathke , A. M. Michel , et al., “Cellular Gene Expression During Hepatitis C Virus Replication as Revealed by Ribosome Profiling,” International Journal of Molecular Sciences 20 (2019): 1321. 10.3390/ijms20061321 PMC6470931 30875926

[91] M. Ripoli , A. D'Aprile , G. Quarato , et al., “Hepatitis C Virus‐Linked Mitochondrial Dysfunction Promotes Hypoxia‐Inducible Factor 1 Alpha‐Mediated Glycolytic Adaptation,” Journal of Virology 84 (2010): 647–660. 10.1128/JVI.00769-09 PMC2798449 19846525

[92] D. Musso and D. J. Gubler , “Zika Virus,” Clinical Microbiology Reviews 29 (2016): 487–524. 10.1128/CMR.00072-15 PMC4861986 27029595

[93] M. I. Giraldo , M. Gonzalez‐Orozco , and R. Rajsbaum , “Pathogenesis of Zika Virus Infection,” Annual Review of Pathology 18 (2023): 181–203. 10.1146/annurev-pathmechdis-031521-034739 PMC10893765 36151059

[94] B. P. Daniels , S. B. Kofman , J. R. Smith , et al., “The Nucleotide Sensor Zbp1 and Kinase Ripk3 Induce the Enzyme Irg1 to Promote an Antiviral Metabolic State in Neurons,” Immunity 50 (2019): 64–76 e4. 10.1016/j.immuni.2018.11.017 PMC6342485 30635240

[95] H. Pang , Y. Jiang , J. Li , et al., “Aberrant NAD (+) Metabolism Underlies Zika Virus‐Induced Microcephaly,” Nature Metabolism 3 (2021): 1109–1124. 10.1038/s42255-021-00437-0 34385701

[96] A. H. Schapira , “Mitochondrial Dysfunction in Neurodegenerative Disorders,” Biochimica et Biophysica Acta (BBA)—Bioenergetics 1366 (1998): 225–233. 10.1016/s0005-2728(98)00115-7 9714816

[97] J. M. Burton , R. Z. Kern , W. Halliday , et al., “Neurological Manifestations of West Nile Virus Infection,” Canadian Journal of Neurological Sciences 31 (2004): 185–193. 10.1017/s0317167100053828 15198442

[98] W. L. Koh and M. L. Ng , “Molecular Mechanisms of West Nile Virus Pathogenesis in Brain Cell,” Emerging Infectious Diseases 11 (2005): 629–632. 10.3201/eid1104.041076 PMC3320339 15829208

[99] P. Mingo‐Casas , A. B. Blazquez , M. Gomez de Cedron , et al., “Glycolytic Shift During West Nile Virus Infection Provides New Therapeutic Opportunities,” Journal of Neuroinflammation 20 (2023): 217. 10.1186/s12974-023-02899-3 PMC10537838 37759218

[100] P. Roingeard , S. Eymieux , J. Burlaud‐Gaillard , et al., “The Double‐Membrane Vesicle (DMV): A Virus‐Induced Organelle Dedicated to the Replication of SARS‐CoV‐2 and Other Positive‐Sense Single‐Stranded RNA Viruses,” Cellular and Molecular Life Sciences 79 (2022): 425. 10.1007/s00018-022-04469-x PMC9287701 35841484

[101] E. J. Snijder , R. Limpens , A. H. de Wilde , et al., “A Unifying Structural and Functional Model of the Coronavirus Replication Organelle: Tracking Down RNA Synthesis,” PLoS Biology 18 (2020): e3000715. 10.1371/journal.pbio.3000715 PMC7302735 32511245

[102] H. J. Shin , W. Lee , K. B. Ku , et al., “Sars‐Cov‐2 Aberrantly Elevates Mitochondrial Bioenergetics to Induce Robust Virus Propagation,” Signal Transduction and Targeted Therapy 9 (2024): 125. 10.1038/s41392-024-01836-x PMC11088672 38734691

[103] A. C. Codo , G. G. Davanzo , L. B. Monteiro , et al., “Elevated Glucose Levels Favor SARS‐CoV‐2 Infection and Monocyte Response Through a Hif‐1alpha/Glycolysis‐Dependent Axis,” Cell Metabolism 32 (2020): 437–446 e5. 10.1016/j.cmet.2020.07.007 PMC7367032 32697943

[104] J. W. Guarnieri , J. M. Dybas , H. Fazelinia , et al., “Core Mitochondrial Genes Are Down‐Regulated During SARS‐CoV‐2 Infection of Rodent and Human Hosts,” Science Translational Medicine 15 (2023): eabq1533. 10.1126/scitranslmed.abq1533 PMC11624572 37556555

[105] B. Miller , A. Silverstein , M. Flores , et al., “Host Mitochondrial Transcriptome Response to SARS‐CoV‐2 in Multiple Cell Models and Clinical Samples,” Scientific Reports 11 (2021): 3. 10.1038/s41598-020-79552-z PMC7794290 33420163

[106] A. Mozzi , M. Oldani , M. E. Forcella , et al., “Sars‐Cov‐2 Orf3c Impairs Mitochondrial Respiratory Metabolism, Oxidative Stress, and Autophagic Flux,” iScience 26 (2023): 107118. 10.1016/j.isci.2023.107118 PMC10265927 37361873

[107] C. Gaebler , Z. Wang , J. C. C. Lorenzi , et al., “Evolution of Antibody Immunity to SARS‐CoV‐2,” Nature 591 (2021): 639–644. 10.1038/s41586-021-03207-w PMC8221082 33461210

[108] S. Gurshaney , A. Morales‐Alvarez , K. Ezhakunnel , et al., “Metabolic Dysregulation Impairs Lymphocyte Function During Severe SARS‐CoV‐2 Infection,” Communications Biology 6 (2023): 374. 10.1038/s42003-023-04730-4 PMC10080180 37029220

[109] I. S. Kim , C. K. Kang , S. J. Lee , et al., “Tracking Antigen‐Specific TCR Clonotypes in Sars‐Cov‐2 Infection Reveals Distinct Severity Trajectories,” Journal of Medical Virology 95 (2023): e29199. 10.1002/jmv.29199 37916645

[110] A. R. Daamen , P. Bachali , K. A. Owen , et al., “Comprehensive Transcriptomic Analysis of Covid‐19 Blood, Lung, and Airway,” Scientific Reports 11 (2021): 7052. 10.1038/s41598-021-86002-x PMC8007747 33782412

[111] E. Dirican , S. T. Savrun , I. E. Aydin , et al., “Analysis of Mitochondrial DNA Cytochrome‐B (Cyb) and ATPase‐6 Gene Mutations in COVID‐19 Patients,” Journal of Medical Virology 94 (2022): 3138–3146. 10.1002/jmv.27704 PMC9088694 35258110

[112] D. Kumari , Y. Singh , S. Singh , et al., “Mitochondrial Pathogenic Mutations and Metabolic Alterations Associated With COVID‐19 Disease Severity,” Journal of Medical Virology 95 no. 2 (2023): e28553. 10.1002/jmv.28553 36832542

[113] Y. Zhang , R. Guo , S. H. Kim , et al., “Sars‐Cov‐2 Hijacks Folate and One‐Carbon Metabolism for Viral Replication,” Nature Communications 12 (2021): 1676. 10.1038/s41467-021-21903-z PMC7960988 33723254

[114] N. Wanner , G. Andrieux , I. M. P. Badia , et al., “Molecular Consequences of SARS‐CoV‐2 Liver Tropism,” Nature Metabolism 4 (2022): 310–319. 10.1038/s42255-022-00552-6 PMC8964418 35347318

[115] H. Yu , L. Yang , Z. Han , et al., “Sars‐Cov‐2 Nucleocapsid Protein Enhances the Level of Mitochondrial Reactive Oxygen Species,” Journal of Medical Virology 95 (2023): e29270. 10.1002/jmv.29270 38047459

[116] D. Dou , R. Revol , H. Östbye , et al., “Influenza A Virus Cell Entry, Replication, Virion Assembly and Movement,” Frontiers in Immunology 9 (2018): 1581. 10.3389/fimmu.2018.01581 PMC6062596 30079062

[117] S. Chua , H. Q. Tan , D. Engelberg , et al., “Alternative Experimental Models for Studying Influenza Proteins, Host‐Virus Interactions and Anti‐Influenza Drugs,” Pharmaceuticals (Basel, Switzerland) 12 (2019): 147. 10.3390/ph12040147 PMC6958409 31575020

[118] J. He , W. B. Qi , L. Wang , et al., “Amaryllidaceae Alkaloids Inhibit Nuclear‐to‐Cytoplasmic Export of Ribonucleoprotein (Rnp) Complex of Highly Pathogenic Avian Influenza Virus H5n1,” Influenza and Other Respiratory Viruses 7 (2013): 922–931. 10.1111/irv.12035 PMC4634243 23136954

[119] J. He , H. Huang , B. Li , et al., “Identification of Cytochrome C Oxidase Subunit 4 Isoform 1 as a Positive Regulator of Influenza Virus Replication,” Frontiers in Microbiology 13 (2022): 862205. 10.3389/fmicb.2022.862205 PMC9343726 35928150

[120] S. Othumpangat , J. D. Noti , and D. H. Beezhold , “Lung Epithelial Cells Resist Influenza A Infection by Inducing the Expression of Cytochrome C Oxidase Vic Which Is Modulated by miRNA 4276,” Virology 468–470 (2014): 256–264. 10.1016/j.virol.2014.08.007 PMC4702256 25203353

[121] A. Ayari , M. Rosa‐Calatrava , S. Lancel , et al., “Influenza Infection Rewires Energy Metabolism and Induces Browning Features in Adipose Cells and Tissues,” Communications Biology 3 (2020): 237. 10.1038/s42003-020-0965-6 PMC7224208 32409640

[122] A. A. Albertini , R. W. Ruigrok , and D. Blondel , “Rabies Virus Transcription and Replication,” Advances in Virus Research 79 (2011): 1–22. 10.1016/B978-0-12-387040-7.00001-9 21601039

[123] T. Alandijany , W. Kammouni , S. K. Roy Chowdhury , et al., “Mitochondrial Dysfunction in Rabies Virus Infection of Neurons,” Journal of Neurovirology 19 (2013): 537–549. 10.1007/s13365-013-0214-6 24277436

[124] W. Kammouni , H. Wood , A. Saleh , et al., “Rabies Virus Phosphoprotein Interacts With Mitochondrial Complex I and Induces Mitochondrial Dysfunction and Oxidative Stress,” Journal of Neurovirology 21 (2015): 370–382. 10.1007/s13365-015-0320-8 25698500

[125] P. K. Harsha , S. Ranganayaki , G. Yale , et al., “Mitochondrial Dysfunction in Rabies Virus‐Infected Human and Canine Brains,” Neurochemical Research 47 (2022): 1610–1636. 10.1007/s11064-022-03556-6 35229271

[126] A. Gholami , R. Kassis , E. Real , et al., “Mitochondrial Dysfunction in Lyssavirus‐Induced Apoptosis,” Journal of Virology 82 (2008): 4774–4784. 10.1128/JVI.02651-07 PMC2346764 18321977

[127] C. Gomez and T. J. Hope , “The Ins and Outs of HIV Replication,” Cellular Microbiology 7 (2005): 621–626. 10.1111/j.1462-5822.2005.00516.x 15839891

[128] E. O. Freed , “HIV‐1 Replication,” Somatic Cell and Molecular Genetics 26 (2001): 13–33. 10.1023/a:1021070512287 12465460

[129] O. Miró , S. López , E. Martínez , et al., “Mitochondrial Effects of HIV Infection on the Peripheral Blood Mononuclear Cells of HIV‐Infected Patients Who Were Never Treated With Antiretrovirals,” Clinical Infectious Diseases 39 (2004): 710–716. 10.1086/423176 15356787

[130] H. C. Lehmann , W. Chen , J. Borzan , et al., “Mitochondrial Dysfunction in Distal Axons Contributes to Human Immunodeficiency Virus Sensory Neuropathy,” Annals of Neurology 69 (2011): 100–110. 10.1002/ana.22150 PMC3051401 21280080

[131] J. S. Ladha , M. K. Tripathy , and D. Mitra , “Mitochondrial Complex I Activity Is Impaired During HIV‐1‐Induced T‐Cell Apoptosis,” Cell Death & Differentiation 12 (2005): 1417–1428. 10.1038/sj.cdd.4401668 15905875

[132] M. K. Tripathy and D. Mitra , “Differential Modulation of Mitochondrial Oxphos System During HIV‐1 Induced T‐Cell Apoptosis: Up Regulation of Complex‐IV Subunit Cox‐II and Its Possible Implications,” Apoptosis 15 (2010): 28–40. 10.1007/s10495-009-0408-9 19771519

[133] S. Svensson Akusjarvi , S. Krishnan , B. B. Jutte , et al., “Peripheral Blood Cd4(+)Ccr6(+) Compartment Differentiates HIV‐1 Infected or Seropositive Elite Controllers From Long‐Term Successfully Treated Individuals,” Communications Biology 5 (2022): 357. 10.1038/s42003-022-03315-x PMC9008025 35418589

[134] I. Clerc , D. A. Moussa , Z. Vahlas , et al., “Entry of Glucose‐ and Glutamine‐Derived Carbons Into the Citric Acid Cycle Supports Early Steps of HIV‐1 Infection in CD4 T Cells,” Nature Metabolism 1 (2019): 717–730. 10.1038/s42255-019-0084-1 PMC7199465 32373781

[135] H. Guo , Q. Wang , K. Ghneim , et al., “Multi‐Omics Analyses Reveal That HIV‐1 Alters CD4(+) T Cell Immunometabolism to Fuel Virus Replication,” Nature Immunology 22 (2021): 423–433. 10.1038/s41590-021-00898-1 PMC8087183 33767427

[136] I. G. Macreadie , D. R. Thorburn , D. M. Kirby , et al., “HIV‐1 Protein VPR Causes Gross Mitochondrial Dysfunction in the Yeast Saccharomyces Cerevisiae,” FEBS Letters 410 (1997): 145–149. 10.1016/s0014-5793(97)00542-5 9237618

[137] M. Ogawa , Y. Takemoto , S. Sumi , et al., “ATP Generation in a Host Cell in Early‐Phase Infection Is Increased by Upregulation of Cytochrome C Oxidase Activity via the P2 Peptide From Human Immunodeficiency Virus Type 1 Gag,” Retrovirology 12 (2015): 97. 10.1186/s12977-015-0224-y PMC4650201 26577226

[138] H. Lecoeur , A. Borgne‐Sanchez , O. Chaloin , et al., “HIV‐1 Tat Protein Directly Induces Mitochondrial Membrane Permeabilization and Inactivates Cytochrome C Oxidase,” Cell Death & Disease 3 (2012): e282. 10.1038/cddis.2012.21 PMC3317353 22419111

[139] B. G. Sousa , N. C. Mebus‐Antunes , L. O. Fernandes‐Siqueira , et al., “Dengue Virus Non‐Structural Protein 3 Inhibits Mitochondrial Respiration by Impairing Complex I Function,” mSphere 9 (2024): e0040624. 10.1128/msphere.00406-24 PMC11288018 38980068

[140] J. Beck and M. Nassal , “Hepatitis B Virus Replication,” World Journal of Gastroenterology 13 (2007): 48–64. 10.3748/wjg.v13.i1.48 PMC4065876 17206754

[141] S. Tsukuda and K. Watashi , “Hepatitis B Virus Biology and Life Cycle,” Antiviral Research 182 (2020): 104925. 10.1016/j.antiviral.2020.104925 32866519

[142] S. Urban , A. Schulze , M. Dandri , et al., “The Replication Cycle of Hepatitis B Virus,” Journal of Hepatology 52 (2010): 282–284. 10.1016/j.jhep.2009.10.031 20056291

[143] M. Nassal , “Hepatitis B Viruses: Reverse Transcription a Different Way,” Virus Research 134 (2008): 235–249. 10.1016/j.virusres.2007.12.024 18339439

[144] P. Honkoop , R. A. de Man , H. R. Scholte , et al., “Effect of Lamivudine on Morphology and Function of Mitochondria in Patients With Chronic Hepatitis B,” Hepatology 26 (1997): 211–215. 10.1002/hep.510260128 9214472

[145] L. Y. Zou , B. Y. Zheng , X. F. Fang , et al., “HBX Co‐Localizes With Cox III in Hl‐7702 Cells to Upregulate Mitochondrial Function and ROS Generation,” Oncology Reports 33 (2015): 2461–2467. 10.3892/or.2015.3852 25778742

[146] D. Li , J. Ding , Z. Chen , et al., “Accurately Mapping the Location of the Binding Site for the Interaction Between Hepatitis B Virus X Protein and Cytochrome C Oxidase III,” International Journal of Molecular Medicine 35 (2015): 319–324. 10.3892/ijmm.2014.2018 PMC4292715 25483779

[147] Y. I. Lee , J. M. Hwang , J. H. Im , et al., “Human Hepatitis B Virus‐X Protein Alters Mitochondrial Function and Physiology in Human Liver Cells,” Journal of Biological Chemistry 279 (2004): 15460–15471. 10.1074/jbc.M309280200 14724286

[148] Y. Li , Y. Zhu , S. Feng , et al., “Macrophages Activated by Hepatitis B Virus Have Distinct Metabolic Profiles and Suppress the Virus via IL‐1beta to Downregulate PPARα and FOXO3,” Cell Reports 38 (2022): 110284. 10.1016/j.celrep.2021.110284 PMC8830375 35081341

[149] B. Damania , S. C. Kenney , and N. Raab‐Traub , “Epstein‐Barr Virus: Biology and Clinical Disease,” Cell 185 (2022): 3652–3670. 10.1016/j.cell.2022.08.026 PMC9529843 36113467

[150] J. M. Silva , C. E. C. Alves , and G. S. Pontes , “Epstein‐Barr Virus: The Mastermind of Immune Chaos,” Frontiers in Immunology 15 (2024): 1297994. 10.3389/fimmu.2024.1297994 PMC10879370 38384471

[151] L. W. Wang , H. Shen , L. Nobre , et al., “Epstein‐Barr‐Virus‐Induced One‐Carbon Metabolism Drives B Cell Transformation,” Cell Metabolism 30 (2019): 539–555.e11. 10.1016/j.cmet.2019.06.003 PMC6720460 31257153

[152] X. Luo , L. Hong , C. Cheng , et al., “Dnmt1 Mediates Metabolic Reprogramming Induced by Epstein‐Barr Virus Latent Membrane Protein 1 and Reversed by Grifolin in Nasopharyngeal Carcinoma,” Cell Death & Disease 9 (2018): 619. 10.1038/s41419-018-0662-2 PMC5966399 29795311

[153] J. Zhang , L. Jia , T. Liu , et al., “Mtorc2‐Mediated Pdhe1alpha Nuclear Translocation Links Ebv‐Lmp1 Reprogrammed Glucose Metabolism to Cancer Metastasis in Nasopharyngeal Carcinoma,” Oncogene 38 (2019): 4669–4684. 10.1038/s41388-019-0749-y PMC6756087 30745576

[154] J. Doorbar , W. Quint , L. Banks , et al., “The Biology and Life‐Cycle of Human Papillomaviruses,” Vaccine 30 (2012): F55–F70, Suppl. 5. 10.1016/j.vaccine.2012.06.083 23199966

[155] S. V. Graham , “The Human Papillomavirus Replication Cycle, and Its Links to Cancer Progression: A Comprehensive Review,” Clinical Science (London, England: 1979) 131 (2017): 2201–2221. 10.1042/CS20160786 28798073

[156] F. Stubenrauch and L. A. Laimins , “Human Papillomavirus Life Cycle: Active and Latent Phases,” Seminars in Cancer Biology 9 (1999): 379–386. 10.1006/scbi.1999.0141 10712884

[157] D. Lai , C. L. Tan , J. Gunaratne , et al., “Localization of HPV‐18 E2 at Mitochondrial Membranes Induces ROS Release and Modulates Host Cell Metabolism,” PLoS ONE 8 (2013): e75625. 10.1371/journal.pone.0075625 PMC3782431 24086592

[158] M. Kirschberg , S. Heuser , G. P. Marcuzzi , et al., “Atp Synthase Modulation Leads to an Increase of Spare Respiratory Capacity in HPV Associated Cancers,” Scientific Reports 10 (2020): 17339. 10.1038/s41598-020-74311-6 PMC7567072 33060693

[159] D. E. Gordon , G. M. Jang , M. Bouhaddou , et al., “A Sars‐Cov‐2 Protein Interaction Map Reveals Targets for Drug Repurposing,” Nature 583 (2020): 459–468. 10.1038/s41586-020-2286-9 PMC7431030 32353859

[160] C. Shang , Z. Liu , Y. Zhu , et al., “Sars‐Cov‐2 Causes Mitochondrial Dysfunction and Mitophagy Impairment,” Frontiers in Microbiology 12 (2021): 780768. 10.3389/fmicb.2021.780768 PMC8770829 35069483

[161] S. Ajaz , M. J. McPhail , K. K. Singh , et al., “Mitochondrial Metabolic Manipulation by Sars‐Cov‐2 in Peripheral Blood Mononuclear Cells of Patients With Covid‐19,” American Journal of Physiology: Cell Physiology 320 (2021): C57–C65. 10.1152/ajpcell.00426.2020 PMC7816428 33151090

[162] M. Hu , K. E. Schulze , R. Ghildyal , et al., “Respiratory Syncytial Virus Co‐Opts Host Mitochondrial Function to Favour Infectious Virus Production,” eLife 8 (2019): e42448. 10.7554/eLife.42448 PMC6598784 31246170

[163] M. Somasundaran , M. L. Zapp , L. K. Beattie , et al., “Localization of HIV RNA in Mitochondria of Infected Cells: Potential Role in Cytopathogenicity,” Journal of Cell Biology 126 (1994): 1353–1360. 10.1083/jcb.126.6.1353 PMC2290946 8089170

[164] F. Real , A. Zhu , B. Huang , et al., “S100a8‐Mediated Metabolic Adaptation Controls HIV‐1 Persistence in Macrophages in Vivo,” Nature Communications 13 (2022): 5956. 10.1038/s41467-022-33401-x PMC9553955 36220814

[165] Z. Yuan , J. R. Petree , F. E. Lee , et al., “Macrophages Exposed to HIV Viral Protein Disrupt Lung Epithelial Cell Integrity and Mitochondrial Bioenergetics via Exosomal MicroRNA Shuttling,” Cell Death & Disease 10 (2019): 580. 10.1038/s41419-019-1803-y PMC6675785 31371699

[166] C. Y. Huang , S. F. Chiang , T. Y. Lin , et al., “HIV‐1 VPR Triggers Mitochondrial Destruction by Impairing Mfn2‐Mediated ER‐Mitochondria Interaction,” PLoS ONE 7 (2012): e33657. 10.1371/journal.pone.0033657 PMC3306277 22438978

[167] A. Macho , M. A. Calzado , L. Jiménez‐Reina , et al., “Susceptibility of HIV‐1‐Tat Transfected Cells to Undergo Apoptosis. Biochemical Mechanisms,” Oncogene 18 (1999): 7543–7551. 10.1038/sj.onc.1203095 10602513

[168] E. Jacotot , L. Ravagnan , M. Loeffler , et al., “The HIV‐1 Viral Protein R Induces Apoptosis via a Direct Effect on the Mitochondrial Permeability Transition Pore,” Journal of Experimental Medicine 191 (2000): 33–46. 10.1084/jem.191.1.33 PMC2195797 10620603

[169] L. O. Fernandes‐Siqueira , J. D. Zeidler , B. G. Sousa , et al., “Anaplerotic Role of Glucose in the Oxidation of Endogenous Fatty Acids During Dengue Virus Infection,” mSphere 3 (2018): e00458–17. 10.1128/mSphere.00458-17 PMC5793041 29404419

[170] L. Chatel‐Chaix , M. Cortese , I. Romero‐Brey , et al., “Dengue Virus Perturbs Mitochondrial Morphodynamics to Dampen Innate Immune Responses,” Cell Host & Microbe 20 (2016): 342–356. 10.1016/j.chom.2016.07.008 PMC7105029 27545046

[171] M. D. Greseth and P. Traktman , “De Novo Fatty Acid Biosynthesis Contributes Significantly to Establishment of a Bioenergetically Favorable Environment for Vaccinia Virus Infection,” PLOS Pathogens 10 (2014): e1004021. 10.1371/journal.ppat.1004021 PMC3961357 24651651

[172] B. Schwer , S. Ren , T. Pietschmann , et al., “Targeting of Hepatitis C Virus Core Protein to Mitochondria Through a Novel C‐Terminal Localization Motif,” Journal of Virology 78 (2004): 7958–7968. 10.1128/JVI.78.15.7958-7968.2004 PMC446112 15254168

[173] R. Fischer , T. Baumert , and H. E. Blum , “Hepatitis C Virus Infection and Apoptosis,” World Journal of Gastroenterology 13 (2007): 4865–4872. 10.3748/wjg.v13.i36.4865 PMC4611765 17828818

[174] C. Fleury , B. Mignotte , and J. L. Vayssière , “Mitochondrial Reactive Oxygen Species in Cell Death Signaling,” Biochimie 84 (2002): 131–141. 10.1016/s0300-9084(02)01369-x 12022944

[175] S. M. Kang , S. J. Kim , J. H. Kim , et al., “Interaction of Hepatitis C Virus Core Protein With Hsp60 Triggers the Production of Reactive Oxygen Species and Enhances TNF‐Alpha‐Mediated Apoptosis,” Cancer Letters 279 (2009): 230–237. 10.1016/j.canlet.2009.02.003 19264393

[176] T. Wang , R. V. Campbell , M. K. Yi , et al., “Role of Hepatitis C Virus Core Protein in Viral‐Induced Mitochondrial Dysfunction,” Journal of Viral Hepatitis 17 (2010): 784–793. 10.1111/j.1365-2893.2009.01238.x PMC2970657 20002299

[177] L. Deng , T. Adachi , K. Kitayama , et al., “Hepatitis C Virus Infection Induces Apoptosis Through a Bax‐Triggered, Mitochondrion‐Mediated, Caspase 3‐Dependent Pathway,” Journal of Virology 82 (2008): 10375–10385. 10.1128/JVI.00395-08 PMC2573220 18768989

[178] D. G. You , H. R. Lee , W. K. Kim , et al., “Hepatitis C Virus P7 Induces Mitochondrial Depolarization of Isolated Liver Mitochondria,” Molecular Medicine Reports 16 (2017): 9533–9538. 10.3892/mmr.2017.7809 29039530

[179] A. Jassey , C. H. Liu , C. A. Changou , et al., “Hepatitis C Virus Non‐Structural Protein 5a (Ns5a) Disrupts Mitochondrial Dynamics and Induces Mitophagy,” Cells 8 (2019): 290. 10.3390/cells8040290 PMC6523690 30934919

[180] C. Yau , J. Z. H. Low , E. S. Gan , et al., “Dysregulated Metabolism Underpins Zika‐Virus‐Infection‐Associated Impairment in Fetal Development,” Cell Reports 37 (2021): 110118. 10.1016/j.celrep.2021.110118 34910902

[181] N. C. Bilz , K. Jahn , M. Lorenz , et al., “Rubella Viruses Shift Cellular Bioenergetics to a More Oxidative and Glycolytic Phenotype With a Strain‐Specific Requirement for Glutamine,” Journal of Virology 92 (2018): e00934–18. 10.1128/JVI.00934-18 PMC6096829 29950419

[182] J. S. Gibbs , D. Malide , F. Hornung , et al., “The Influenza A Virus Pb1‐F2 Protein Targets the Inner Mitochondrial Membrane via a Predicted Basic Amphipathic Helix That Disrupts Mitochondrial Function,” Journal of Virology 77 (2003): 7214–7224. 10.1128/JVI.77.13.7214-7224.2003 PMC164823 12805420

[183] H. Yamada , R. Chounan , Y. Higashi , et al., “Mitochondrial Targeting Sequence of the Influenza a Virus Pb1‐F2 Protein and Its Function in Mitochondria,” FEBS Letters 578 (2004): 331–336. 10.1016/j.febslet.2004.11.017 15589841

[184] M. Derakhshan , M. M. Willcocks , M. A. Salako , et al., “Human Herpesvirus 1 Protein Us3 Induces an Inhibition of Mitochondrial Electron Transport,” Journal of General Virology 87 (2006): 2155–2159. 10.1099/vir.0.81949-0 16847111

[185] S. Takada , Y. Shirakata , N. Kaneniwa , et al., “Association of Hepatitis B Virus X Protein With Mitochondria Causes Mitochondrial Aggregation at the Nuclear Periphery, Leading to Cell Death,” Oncogene 18 (1999): 6965–6973. 10.1038/sj.onc.1203188 10597295

[186] Z. Rahmani , K. W. Huh , R. Lasher , et al., “Hepatitis B Virus X Protein Colocalizes to Mitochondria With a Human Voltage‐Dependent Anion Channel, Hvdac3, and Alters Its Transmembrane Potential,” Journal of Virology 74 (2000): 2840–2846. 10.1128/jvi.74.6.2840-2846.2000 PMC111774 10684300

[187] S. L. McClain , A. J. Clippinger , R. Lizzano , et al., “Hepatitis B Virus Replication Is Associated With an HBx‐Dependent Mitochondrion‐Regulated Increase in Cytosolic Calcium Levels,” Journal of Virology 81 (2007): 12061–12065. 10.1128/JVI.00740-07 PMC2168786 17699583

[188] B. Yang and M. J. Bouchard , “The Hepatitis B Virus X Protein Elevates Cytosolic Calcium Signals by Modulating Mitochondrial Calcium Uptake,” Journal of Virology 86 (2012): 313–327. 10.1128/JVI.06442-11 PMC3255901 22031934

[189] D. Giosa , D. Lombardo , C. Musolino , et al., “Mitochondrial DNA Is a Target of HBV Integration,” Communications Biology 6 (2023): 684. 10.1038/s42003-023-05017-4 PMC10318008 37400627

[190] S. J. Kim , M. Khan , J. Quan , et al., “Hepatitis B Virus Disrupts Mitochondrial Dynamics: Induces Fission and Mitophagy to Attenuate Apoptosis,” PLOS Pathogens 9 (2013): e1003722. 10.1371/journal.ppat.1003722 PMC3855539 24339771

[191] Y. S. Yoo , Y. J. Park , H. S. Lee , et al., “Mitochondria Ubiquitin Ligase, March5 Resolves Hepatitis B Virus X Protein Aggregates in the Liver Pathogenesis,” Cell Death & Disease 10 (2019): 938. 10.1038/s41419-019-2175-z PMC6901512 31819032

[192] J. Camps , S. Iftimie , A. Jiménez‐Franco , et al., “Metabolic Reprogramming in Respiratory Viral Infections: A Focus on Sars‐Cov‐2, Influenza, and Respiratory Syncytial Virus,” Biomolecules 15 (2025): 1027. 10.3390/biom15071027 PMC12292877 40723899

[193] J. Y. Wang , J. Q. Li , Y. M. Xiao , et al., “Triphenylphosphonium (TPP)‐Based Antioxidants: A New Perspective on Antioxidant Design,” ChemMedChem 15 (2020): 404–410. 10.1002/cmdc.201900695 32020724

[194] M. J. Rossman , J. R. Santos‐Parker , C. A. C. Steward , et al., “Chronic Supplementation With a Mitochondrial Antioxidant (MitoQ) Improves Vascular Function in Healthy Older Adults,” Hypertension 71 (2018): 1056–1063. 10.1161/HYPERTENSIONAHA.117.10787 PMC5945293 29661838

[195] W. W. Wheaton , S. E. Weinberg , R. B. Hamanaka , et al., “Metformin Inhibits Mitochondrial Complex I of Cancer Cells to Reduce Tumorigenesis,” eLife 3 (2014): e02242. 10.7554/eLife.02242 PMC4017650 24843020

[196] T. Defresne , R. Suspène , and J. P. Vartanian , “Cellular Titanomachy: Viral Forces Clash With Mitochondrial Power,” Annual Review of Virology 12 (2025): 157–178. 10.1146/annurev-virology-092623-090901 40998741

[197] J. Foo , G. Bellot , S. Pervaiz , et al., “Mitochondria‐Mediated Oxidative Stress During Viral Infection,” Trends in Microbiology 30 (2022): 679–692. 10.1016/j.tim.2021.12.011 35063304

[198] S. E. Pérez , M. Gooz , and E. N. Maldonado , “Mitochondrial Dysfunction and Metabolic Disturbances Induced by Viral Infections,” Cells 13 (2024): 1789. 10.3390/cells13211789 PMC11545457 39513896

[199] N. Kozhukhar and M. F. Alexeyev , “Off‐Target Effects of Mitochondrial Oxidative Phosphorylation Inhibitors Are Common and Can Compromise the Viability of Cultured Cells,” Mitochondrial Communications 3 (2025): 99–108. 10.1016/j.mitoco.2025.12.001 PMC12834556 41602440

[200] A. L. Arthurs , D. J. Keating , B. W. Stringer , et al., “The Suitability of Glioblastoma Cell Lines as Models for Primary Glioblastoma Cell Metabolism,” Cancers 12 (2020): 3722. 10.3390/cancers12123722 PMC7764800 33322454

[201] J. Tisoncik‐Go , D. J. Gasper , J. E. Kyle , et al., “Integrated Omics Analysis of Pathogenic Host Responses During Pandemic H1n1 Influenza Virus Infection: The Crucial Role of Lipid Metabolism,” Cell Host & Microbe 19 (2016): 254–266. 10.1016/j.chom.2016.01.002 PMC5271177 26867183

[202] D. A. Dean , L. Klechka , E. Hossain , et al., “Spatial Metabolomics Reveals Localized Impact of Influenza Virus Infection on the Lung Tissue Metabolome,” mSystems 7 (2022): e0035322. 10.1128/msystems.00353-22 PMC9426520 35730946

[203] A. Hafner , R. L. Mokry , J. G. Purdy , et al., “Metabolic Hijackers: How Viral Proteins Redefine Host Cell Landscapes,” Journal of Virology 100 (2026): e0055625. 10.1128/jvi.00556-25 PMC12911867 41511084

[204] K. Wheeler , C. Gosmanov , M. J. Sandoval , et al., “Frontiers in Mass Spectrometry‐Based Spatial Metabolomics: Current Applications and Challenges in the Context of Biomedical Research,” Trends in Analytical Chemistry 175 (2024): 117713. 10.1016/j.trac.2024.117713 PMC11905388 40094101

[205] J. L. Jacobs and C. B. Coyne , “Mechanisms of Mavs Regulation at the Mitochondrial Membrane,” Journal of Molecular Biology 425 (2013): 5009–5019. 10.1016/j.jmb.2013.10.007 PMC4562275 24120683

[206] E. J. Gane , F. Weilert , D. W. Orr , et al., “The Mitochondria‐Targeted Anti‐Oxidant Mitoquinone Decreases Liver Damage in a Phase II Study of Hepatitis C Patients,” Liver International 30 (2010): 1019–1026. 10.1111/j.1478-3231.2010.02250.x 20492507

